# Alcohol intake and risk of stomach cancer: a pooled analysis of 20 cohorts

**DOI:** 10.1093/jncics/pkag073

**Published:** 2026-07-24

**Authors:** Harindra Jayasekara, Molin Wang, Yang Peng, Sarah K Abe, Christian C Abnet, Hui Cai, Andrew T Chan, Mridul Datta, A Heather Eliassen, Yu-Tang Gao, Edward L Giovannucci, Mayo Hirabayashi, Tao Hou, Wen-Yi Huang, Ugonna Ihenacho, Manami Inoue, Woon-Puay Koh, Susanna C Larsson, Linda M Liao, Erikka Loftfield, Marjorie L McCullough, Anthony B Miller, Lorelei A Mucci, Gwen A Murphy, Jin Young Park, Song-Yi Park, Anna Prizment, Kim Robien, Thomas E Rohan, Norie Sawada, Marissa M Shams-White, Xiao-Ou Shu, Rashmi Sinha, Mingyang Song, Meir J Stampfer, Yangbo Sun, Anne Tjønneland, Caroline Y Um, Piet A van den Brandt, Renwei Wang, Jean Wactawski-Wende, Emily White, Lynne Wilkens, Alicja Wolk, Jian-Min Yuan, Robin Room, Andrew Haydon, Robert J MacInnis, M Constanza Camargo, Graham G Giles, Roger L Milne, Pietro Ferrari, Stephanie A Smith-Warner

**Affiliations:** Cancer Epidemiology Division, Cancer Council Victoria, Melbourne, Victoria, Australia; Centre for Epidemiology and Biostatistics, Melbourne School of Population and Global Health, The University of Melbourne, Melbourne, Victoria, Australia; School of Public Health and Preventive Medicine, Faculty of Medicine, Nursing and Health Sciences, Monash University, Clayton, Victoria, Australia; Department of Epidemiology, Harvard T.H. Chan School of Public Health, Boston, MA, United States; Department of Biostatistics, Harvard T.H. Chan School of Public Health, Boston, MA, United States; Channing Division of Network Medicine, Department of Medicine, Brigham and Women’s Hospital and Harvard Medical School, Boston, MA, United States; Cancer Epidemiology Division, Cancer Council Victoria, Melbourne, Victoria, Australia; School of Public Health, The University of Queensland, Queensland, Australia; Division of Prevention, National Cancer Center Institute for Cancer Control, Tokyo, Japan; Division of Cancer Epidemiology and Genetics, National Cancer Institute, National Institutes of Health, Rockville, MD, United States; Division of Epidemiology, Department of Medicine, Vanderbilt University School of Medicine, Nashville, TN, United States; Channing Division of Network Medicine, Department of Medicine, Brigham and Women’s Hospital and Harvard Medical School, Boston, MA, United States; Clinical and Translational Epidemiology Unit, Massachusetts General Hospital, Boston, MA, United States; Department of Food Science and Human Nutrition, Iowa State University, Ames, IA, United States; Department of Epidemiology, Harvard T.H. Chan School of Public Health, Boston, MA, United States; Channing Division of Network Medicine, Department of Medicine, Brigham and Women’s Hospital and Harvard Medical School, Boston, MA, United States; Department of Nutrition, Harvard T. H. Chan School of Public Health, Boston, MA, United States; Shanghai Cancer Institute, Shanghai, China; Department of Epidemiology, Harvard T.H. Chan School of Public Health, Boston, MA, United States; Department of Nutrition, Harvard T. H. Chan School of Public Health, Boston, MA, United States; Division of Prevention, National Cancer Center Institute for Cancer Control, Tokyo, Japan; Department of Nutrition, Harvard T. H. Chan School of Public Health, Boston, MA, United States; Division of Cancer Epidemiology and Genetics, National Cancer Institute, National Institutes of Health, Rockville, MD, United States; Department of Population and Public Health Sciences, Keck School of Medicine, University of Southern California, Los Angeles, CA, United States; Division of Prevention, National Cancer Center Institute for Cancer Control, Tokyo, Japan; Healthy Longevity Translational Research Programme, Yong Loo Lin School of Medicine, National University of Singapore, Singapore, Singapore; Unit of Cardiovascular and Nutritional Epidemiology, Institute of Environmental Medicine, Karolinska Institutet, Stockholm, Sweden; Division of Cancer Epidemiology and Genetics, National Cancer Institute, National Institutes of Health, Rockville, MD, United States; Division of Cancer Epidemiology and Genetics, National Cancer Institute, National Institutes of Health, Rockville, MD, United States; Department of Population Science, American Cancer Society, Atlanta, GA, United States; Department of Public Health Sciences, University of Toronto, Canada; Department of Epidemiology, Harvard T.H. Chan School of Public Health, Boston, MA, United States; Discovery Sciences, American Cancer Society, Atlanta, GA, United States; Cancer Screening and Prevention Research Group, Department of Surgery & Cancer, Imperial College London, London, United Kingdom; Early Detection, Prevention, and Infections Branch, International Agency for Research on Cancer, Lyon Cedex, France; Population Sciences in the Pacific Program, University of Hawaii Cancer Center, Honolulu, HI, United States; Department of Laboratory Medicine and Pathology, University of Minnesota Medical School, Minneapolis, MN, United States; University of Minnesota Masonic Cancer Center, Minneapolis, MN, United States; Department of Exercise and Nutrition Sciences, Milken Institute School of Public Health, George Washington University, Washington, DC, United States; Department of Epidemiology and Population Health, Albert Einstein College of Medicine, Bronx, NY, United States; Division of Cohort Research, National Cancer Center Institute for Cancer Control, Tokyo, Japan; Department of Population Science, American Cancer Society, Atlanta, GA, United States; Division of Epidemiology, Department of Medicine, Vanderbilt University School of Medicine, Nashville, TN, United States; Division of Cancer Epidemiology and Genetics, National Cancer Institute, National Institutes of Health, Rockville, MD, United States; Department of Epidemiology, Harvard T.H. Chan School of Public Health, Boston, MA, United States; Department of Epidemiology, Harvard T.H. Chan School of Public Health, Boston, MA, United States; Channing Division of Network Medicine, Department of Medicine, Brigham and Women’s Hospital and Harvard Medical School, Boston, MA, United States; Department of Nutrition, Harvard T. H. Chan School of Public Health, Boston, MA, United States; Department of Preventive Medicine, The University of Tennessee Health Science Center, Memphis, TN, United States; Diet, Cancer and Health, Danish Cancer Institute, Copenhagen, Denmark; Department of Population Science, American Cancer Society, Atlanta, GA, United States; Department of Epidemiology, Maastricht University, Maastricht, The Netherlands; Hillman Cancer Center, University of Pittsburgh, Pittsburgh, PA, United States; University at Buffalo Department of Epidemiology and Environmental Health, University at Buffalo, Buffalo, NY, United States; Fred Hutchinson Cancer Research Center, Seattle, WA, United States; Population Sciences, University of Hawaiʻi Cancer Center, Honolulu, HI, United States; Unit of Cardiovascular and Nutritional Epidemiology, Institute of Environmental Medicine, Karolinska Institutet, Stockholm, Sweden; UPMC Hillman Cancer Center and Department of Epidemiology, School of Public Health, University of Pittsburgh, Pittsburgh, PA, United States; Centre for Alcohol Policy Research, La Trobe University, Melbourne, Australia; Social Research Centre on Alcohol and Drugs, Stockholm University, Stockholm, Sweden; Department of Medical Oncology, Alfred Hospital, Melbourne, Victoria, Australia; Cancer Epidemiology Division, Cancer Council Victoria, Melbourne, Victoria, Australia; Centre for Epidemiology and Biostatistics, Melbourne School of Population and Global Health, The University of Melbourne, Melbourne, Victoria, Australia; Division of Cancer Epidemiology and Genetics, National Cancer Institute, National Institutes of Health, Rockville, MD, United States; Cancer Epidemiology Division, Cancer Council Victoria, Melbourne, Victoria, Australia; Centre for Epidemiology and Biostatistics, Melbourne School of Population and Global Health, The University of Melbourne, Melbourne, Victoria, Australia; Precision Medicine, School of Clinical Sciences at Monash Health, Monash University, Clayton, Victoria, Australia; Cancer Epidemiology Division, Cancer Council Victoria, Melbourne, Victoria, Australia; Centre for Epidemiology and Biostatistics, Melbourne School of Population and Global Health, The University of Melbourne, Melbourne, Victoria, Australia; Precision Medicine, School of Clinical Sciences at Monash Health, Monash University, Clayton, Victoria, Australia; Nutrition and Metabolism Branch, International Agency for Research on Cancer, Lyon Cedex, France; Department of Epidemiology, Harvard T.H. Chan School of Public Health, Boston, MA, United States; Department of Nutrition, Harvard T. H. Chan School of Public Health, Boston, MA, United States

## Abstract

**Background:**

Stomach cancer presents complex etiological heterogeneity. Ethanol in alcoholic beverages and its metabolite acetaldehyde are carcinogens causally linked to several cancers, but their role in gastric carcinogenesis has not been established. We analyzed harmonized, individual-level prospective data to examine associations between alcohol intake and risk of stomach cancer and its subtypes.

**Methods:**

In total, 2 009 951 participants in 20 cohorts (mean follow-up = 9 to 29 years) within the Pooling Project of Prospective Studies of Diet and Cancer (n = 8357 incident invasive gastric adenocarcinomas) were included. We used Cox regression to assess associations between alcohol intake and risk of stomach cancer overall and by anatomical and histological subtype and population subgroup, adjusting for confounders.

**Results:**

Evidence for an association between alcohol intake and overall stomach cancer risk was weak (hazard ratio, HR, for ≥30 vs 0.1-<5 g/day: 1.06 [95% confidence interval, CI = 0.96 to 1.16], *P*_between-studies heterogeneity_ = .43). Positive associations with stomach cancer risk were observed in never smokers (HR, for ≥30 vs 0.1-<5 g/day: 1.20 [95% CI = 1.02 to 1.42]; *P*_interaction_ = .02) and for Asian studies (HR, 1.21 [95% CI = 1.02 to 1.42]; *P*_interaction_ = .01). Modest increased risks were observed for noncardia cancers such as those of the fundus, body, and greater curvature, but not distally located noncardia cancers. HRs did not differ materially between diffuse- and intestinal-type cancers (*P*_heterogeneity_ > .05).

**Conclusion:**

There was little evidence of an overall association between alcohol intake and stomach cancer risk, although modest positive associations were observed among never smokers and in Asian

## Introduction

Stomach cancer is the fifth most common cancer globally and the fifth leading cause of death from cancer^1^ as the diagnosis is often made when the disease is at an advanced stage.^2^ Adenocarcinomas make up more than 90% of stomach cancers and are a heterogeneous group in terms of etiology, pathogenesis, molecular pathology, and prognosis.^3^^,^^4^  *Helicobacter pylori* infection and smoking are established causes, but epidemiological evidence remains equivocal for other modifiable lifestyle-related factors.^5^ Ethanol in alcoholic beverages and its toxic metabolite acetaldehyde are classified as group 1 carcinogens.^6^ Alcohol consumption is causally linked to cancers of the oral cavity, pharynx, larynx, esophagus (squamous-cell carcinoma), liver, colorectum, and female breast.^7^^,^^8^ Even though it is highly plausible,^9^ a role for alcohol use in the etiology of stomach cancer has not been established thus far. Individual observational studies usually lack sufficient statistical power; results from meta-analyses of cohort studies have been inconsistent or weak, combined results using different reference groups, displayed significant evidence of small study bias, and did not adjust for *H. pylori* infection.^5^

To address these limitations, we pooled individual-level data from 20 cohorts to assemble the world’s largest pooled prospective dataset to date, with the aim of assessing associations between alcohol intake and the risk of stomach cancer and its anatomical subsites, histology subtypes, overall and across population subgroups.

## Materials and methods

### Study population

This analysis includes 20 prospective cohort studies that recruited participants from 14 countries (Table 1)^10-27^ within the Pooling Project of Prospective Studies of Diet and Cancer (DCPP)^28^ Working Group of the National Cancer Institute Cohort Consortium.^29^ Details on inclusion/exclusion criteria for cohorts and participants are provided in Supplementary Section 1. The cohort studies, the consortium, and participating registries (as required) were approved by their respective institutional review boards.

**Table 1.** Characteristics of the cohort studies included in the pooled analyses of alcohol intake and stomach cancer risk.

Study (country/continent)	**Baseline Cohort size** ^a^	Baseline year	Mean follow-up time (yrs)	Baseline age range (yrs)	% Women	% Ever Smokers	**No. of cases** ^b^				Gastric cardia: Noncardia ratio	Alcohol intake for women		Alcohol intake for men	
							Total (n)	Gastric cardia (n, %)	Noncardia (n, %)	Overlapping/unspecified (n, %)		% Drinkers	Median (10th-90th percentile) among drinkers (g/day)	% Drinkers	Median (10th-90th percentile) among drinkers (g/day)
Canadian National Breast Screening Study (Canada)	48 778	1980-85	22	40 to 59	100	50	97	23 (24)	33 (34)	41 (42)	1:1.4	77	7 (1 to 28)	–	
Cancer Prevention Study II Nutrition Cohort (USA)	140 491	1992-93	16	50 to 74	53	56	269	127 (47)	98 (36)	44 (17)	1:0.8	52	4 (1 to 24)	65	10 (1 to 46)
Cohort of Swedish Men (Sweden)	45 219	1997	19	45 to 79	0	63	175	62 (35)	63 (36)	50 (29)	1:1.0	–	–	92	9 (2 to 24)
European Prospective Investigation into Cancer and Nutrition (Europe)	414 505	1991 to 2001	15	35 to 70	68	49	836	221 (26)	318 (38)	298 (36)	1:1.4	83	6 (0.5 to 25)	93	14 (2 to 51)
Health Professionals Follow-up Study (USA)	47 766	1986-87	26	40 to 75	0	52	145*	–	–	–	–	–	–	76	10 (2 to 37)
Iowa Women’s Health Study (USA)	34 578	1986	22	55 to 69	100	34	69	22 (32)	33 (48)	14 (20)	1:1.5	45	3 (1 to 21)	–	–
Japan Public Health Center-based Prospective Study I (Japan)	39 032	1995	18	40 to 59	53	33	801**	52 (7)	636 (79)	99 (12)	1:12.2	17	5 (1 to 23)	76	36 (5 to 74)
Japan Public Health Center-based Prospective Study II (Japan)	49 183	1995	15	40 to 69	53	31	805	41 (5)	586 (73)	178 (22)	1:14.3	18	5 (1 to 26)	72	33 (4 to 84)
Melbourne Collaborative Cohort Study (Australia)	36 085	1990-94	24	40 to 69	61	41	173	41 (24)	89 (51)	43 (25)	1:2.2	53	9 (1 to 30)	74	17 (2 to 56)
Multiethnic Cohort Study (USA)	169 926	1993-96	20	45 to 75	54	55	1260	188 (15)	806 (64)	266 (21)	1:4.3	37	3 (0.4 to 28)	62	11 (1 to 51)
Netherlands Cohort Study (Netherlands)	120 852	1986	17	55 to 69	46	67	768	197 (26)	346 (45)	225 (29)	1:1.8	68	4 (1.23)	86	13 (2 to 39)
NIH-AARP Diet and Health Study (USA)	490 722	1995-97	16	50 to 71	41	61	1183	567 (48)	396 (34)	220 (19)	1:0.7	70	2 (0.4 to 20)	79	7 (1 to 48)
Nurses’ Health Study (USA)	68 447	1986-88	29	30 to 55	100	56	99*	–	–	–	–	64	5 (1 to 28)	–	–
Prostate, Lung, Colorectal, and Ovarian Cancer Screening Trial (USA)^c^	49 098	1993 to 2001	9	55 to 74	0	62	122	55 (45)	41 (34)	26 (21)	1:0.7	–	–	76	7 (1 to 42)
Shanghai Cohort Study (China)	16 339	1986-89	25	45 to 64	0	57	434	83 (19)	276 (64)	75 (17)	1:3.3	–	–	42	22 (4 to 65)
Shanghai Men’s Health Study (China)	61 060	2002-06	12	40 to 74	0	70	516	46 (9)	372 (72)	98 (19)	1:8.1	–	–	32	26 (9 to 70)
Singapore Chinese Health Study (Singapore)^d^	27 175	1993-98	14	45 to 74	0	58	334	52 (16)	196 (59)	86 (26)	1:3.8	–	–	32	4 (0.4 to 28)
Swedish Mammography Cohort (Sweden)	34 982	1987-90	19	40 to 76	100	45	95	16 (17)	44 (46)	35 (37)	1:2.8	82	3 (0.4 to 10)	–	–
VITamins and Lifestyle (VITAL) Study: Cohort Study of Dietary Supplements and Cancer Risk (USA)^c^	30 168	2000-02	10	50 to 76	0	60	61	41 (67)	11 (18)	9 (15)	1:0.3	–	–	71	10 (1 to 40)
Women’s Health Initiative (USA)	85 545	1993-98	19	50 to 79	100	48	115	33 (29)	56 (49)	26 (22)	1:1.7	58	5 (1 to 24)	–	–
Total	2009 951						8357	1867 (22)	4400 (53)	1832 (22)					

a Cohort size was calculated after applying study-specific exclusion criteria and further excluding participants with history of cancer diagnosis at baseline (except for nonmelanoma skin cancer), energy intakes beyond 3 standard deviations of their log_e_-transformed study specific mean energy intake (except in the Shanghai Cohort Study, which did not include a comprehensive dietary assessment), missing data on alcohol intake, or with alcohol intake >200 g/day; the Netherlands Cohort Study was analyzed as a case-cohort study and the above exclusions were not applied to their baseline cohort size.

b Gastric cardia: International Classification of Diseases (ICD) 9/ICD 10 codes 151.0/C16.0; noncardia: 151.1 to 151.6/C16.1-C16.6; and overlapping/unspecified tumors: 151.8/C16.8 and 151.9/C16.9; *All are missing subsite; **n = 14 missing subsite.

c Men only as <50 incident cases of adenocarcinoma of the stomach were documented for women.

d Men only as ≤10% of women were alcohol drinkers.

### Assessment of dietary and nondietary factors

Alcoholic beverage consumption was assessed at or near study enrollment, using a food frequency questionnaire (FFQ) or diet history covering the past year (see Supplementary Section 2 for more details). Each study measured usual consumption of individual alcoholic beverages (beer, wine, spirits). For each beverage, daily alcohol intake in grams was calculated based on the frequency of consumption, the ethanol content of the beverage, and the average quantity consumed. Study-specific conversion factors for the ethanol content of each beverage were used. We computed total alcohol consumption by summing alcohol intake as grams per day from each alcoholic beverage. The correlation coefficients between intakes estimated from the FFQ/diet history and the reference method generally exceeded 0.7 for alcohol intake across studies.^28^^,^^30-42^

Demographic details, medical history, and lifestyle factors were collected at enrollment and/or at the same time that diet was assessed and harmonized across studies (see Supplementary Section 2 for more details).

Six cohorts assessed *H. pylori* serostatus in nested case-control studies on stomach cancer^13^^,^^16^^,^^17^^,^^21^^,^^23^ (described in more detail in Supplementary Section 3).

### Outcome

Incident invasive adenocarcinoma of the stomach (coded as 151 according to the ninth revision of the International Classification of Diseases [ICD]^43^ and C16 according to the 10th revision of the ICD^44^) was defined as the primary outcome of interest. We defined subtypes of stomach cancer based on anatomical site (gastric cardia, noncardia, and overlapping/unspecified lesions of the stomach; and fundus, body, greater curvature, lesser curvature, pyloric antrum, and pylorus;^45^ see Table 4 footnote for ICD codes) and histology (diffuse type, intestinal type, and mixed/other/unknown types; see Table S1 for International Classification of Diseases for Oncology [ICD-O-3]^46^ codes).

Cancer outcomes were ascertained by self-report and medical record reviews,^14^^,^^27^ linkage to cancer registries,^10^^,^^12^^,^^15^^,^^17-20^^,^^23-25^ or both methods.^11^^,^^13^^,^^16^^,^^21^^,^^22^^,^^26^ Some studies additionally identified cancer outcomes using linkage to mortality registries.^11^^,^^12^^,^^14^^,^^15^^,^^17^^,^^22^^,^^23^^,^^25^

### Statistical analysis

We analyzed the primary participant-level data in each cohort. Cohorts including women and men were analyzed as two separate studies. The Netherlands Cohort Study^19^ was analyzed as a case-cohort study.^47^ Details on exclusion criteria for participants are provided in Supplementary Section 4. The association between alcohol intake and risk of total stomach cancer was evaluated using Cox regression.^48^ Alcohol intake was modeled using categories defined by common absolute intake cut points (abstainers defined as <0.1, 0.1-<5 [ref], 5-<15, 15-<30, ≥30 g/day). The category of 0.1-<5 g/day was designated as the reference, due to the potential inclusion of former drinkers, including those who stopped drinking for health reasons, within the nondrinker category, which could introduce misclassification bias. A continuous variable was created using the median of each intake category to assess the significance of the trend in alcohol drinkers with the Wald test *P* value. For each participant, person-years of follow-up was defined from baseline questionnaire return (here defined as when alcohol intake was assessed) to diagnosis of any incident cancer, death, loss to follow-up, or administrative end of follow-up, whichever occurred first. We jointly stratified models by age at baseline and calendar year of questionnaire return to account for age, calendar time, and time since study entry, and by study center (for the European Prospective Investigation into Cancer and Nutrition^13^). Confounding variables were determined using a causal diagram (directed acyclic graph) and existing evidence and included race and ethnicity, education, cigarette smoking (status, amount smoked, duration), body mass index (BMI), red meat intake, fruit intake, and total energy intake, and an indicator variable to denote alcohol intake as any or none (see details in Table 2 footnote).

**Table 2.** Pooled hazard ratios (HR) and 95% confidence intervals (95% CI) for alcohol intake and stomach cancer risk.

		**Per 10 g/day increment** ^h^	Alcohol intake categories (g/day)					***P* trend** ^b^	***P*, Test for between-studies heterogeneity** ^c^	*I* ^2^ statistic, Highest category	***P*, Test for between-studies heterogeneity due to sex** ^d^
			**Abstention** ^a^	0.1-<5	5-<15	15-<30	≥30				
All											
	No. of cases (%)	8357 (100)	3255 (39.0)	1669 (20.0)	1185 (14.2)	974 (11.6)	1274 (15.2)				
Age adjusted	HR (95% CI)^e^	1.01 (1.00 to 1.03)	1.12 (1.04 to 1.21)	1.00 (ref)	0.94 (0.87 to 1.02)	1.03 (0.91 to 1.17)	1.16 (1.05 to 1.28)	.002	.33	10%	.97
Age and smoking	HR (95% CI)^f^	0.99 (0.97 to 1.01)	1.12 (1.04 to 1.21)	1.00 (ref)	0.92 (0.85 to 1.00)	0.98 (0.87 to 1.11)	1.04 (0.94 to 1.15)	.36	.36	8%	.81
Multivariable	HR (95% CI)^g^	1.00 (0.98 to 1.02)	1.08 (1.01 to 1.16)	1.00 (ref)	0.94 (0.87 to 1.02)	1.02 (0.91 to 1.14)	1.06 (0.96 to 1.16)	.25	.43	3%	.83
Women											
	No. of cases (%)	2406 (100)	1279 (53.2)	648 (26.9)	282 (11.7)	114 (4.7)	83 (3.5)				
Age adjusted	HR (95% CI)^e^	0.96 (0.91 to 1.01)	1.12 (0.97 to 1.30)	1.00 (ref)	0.88 (0.76 to 1.02)	0.83 (0.62 to 1.11)	1.16 (0.92 to 1.48)	.60	.50	0%	–
Age and smoking	HR (95% CI)^f^	0.93 (0.88 to 0.98)	1.13 (0.97 to 1.31)	1.00 (ref)	0.86 (0.74 to 0.99)	0.78 (0.58 to 1.04)	1.01 (0.79 to 1.29)	.56	.55	0%	–
Multivariable	HR (95% CI)^g^	0.96 (0.92 to 1.00)	1.06 (0.92 to 1.23)	1.00 (ref)	0.89 (0.77 to 1.02)	0.83 (0.62 to 1.10)	1.08 (0.85 to 1.38)	.96	.55	0%	–
Men											
	No. of cases (%)	5951 (100)	1976 (33.2)	1021 (17.2)	903 (15.2)	860 (14.4)	1191 (20.0)				
Age adjusted	HR (95% CI)^e^	1.02 (1.01 to 1.04)	1.12 (1.02 to 1.22)	1.00 (ref)	0.97 (0.88 to 1.07)	1.11 (0.99 to 1.24)	1.15 (1.02 to 1.30)	.002	.20	24%	–
Age and smoking	HR (95% CI)^f^	1.01 (0.99 to 1.03)	1.12 (1.03 to 1.22)	1.00 (ref)	0.95 (0.87 to 1.05)	1.06 (0.95 to 1.18)	1.04 (0.92 to 1.18)	.22	.21	23%	–
Multivariable	HR (95% CI)^g^	1.01 (0.99 to 1.03)	1.09 (1.00 to 1.19)	1.00 (ref)	0.97 (0.88 to 1.07)	1.08 (0.98 to 1.19)	1.05 (0.93 to 1.18)	.25	.27	17%	–

a Abstention was defined as <0.1 g/day.

b *P* value, test for trend was calculated in drinkers (intake ≥0.1 g/day) using the Wald test statistic with the median of each intake category fit as a continuous variable.

c *P* value, test for between-studies heterogeneity is given for ≥30 g/day category of alcohol intake.

d *P* value, test for between-studies heterogeneity due to sex is given for ≥30 g/day category of alcohol intake.

e Sex-specific cohorts were analyzed. Stratified by age in years, year of questionnaire return, and center (for European Prospective Investigation into Cancer and Nutrition).

f Further adjusted for cigarette smoking status (never, past, current), amount smoked (cigarettes/day, continuous), and duration of cigarette smoking (years, continuous).

g Further adjusted for race and ethnicity (Asian, Black, Hispanic, White, other), education (<high school, high school, >high school), body mass index (kg/m^2^, continuous), red meat intake (g/day, tertiles), fruit intake (g/day, tertiles), and total energy intake (kcal/day, continuous).

h Analyses for continuous alcohol intake were further adjusted for an indicator variable identifying alcohol drinking status.

To examine associations between alcohol intake and risk of total stomach cancer, we initially analyzed each study separately. The study-specific hazard ratios (HRs) were pooled using random-effects models^49^ weighted by the sum of the inverse of the variance and the estimated between-studies variance components. Between-studies heterogeneity was evaluated using the *Q*-statistic^49^ and *I*^2^ statistic,^50^ and heterogeneity by sex was examined using a meta-regression model.^51^

We repeated the analysis for total stomach cancer using aggregated data where the individual level data from each study were combined into a single dataset. All other analyses were conducted using the aggregated data set owing to the small case numbers within most studies and included a consistent set of cohorts throughout. In all aggregated analyses, we additionally stratified participants by study. To evaluate potential effect modification, we conducted analyses in which participants were stratified by smoking status (never, past, current), geographic region (Asia, Europe/Australia, North America), BMI (<25, ≥25 kg/m^2^), or follow-up time (<5, 5-<10, ≥10 years). We tested whether the association with alcohol intake varied by each stratification factor using the likelihood ratio test for the cross-product terms of alcohol intake modeled continuously and the potential effect modifier modeled categorically.^28^^,^^52^ We tested whether associations differed by attained age (<65, 65-<75, ≥75 years) and across subtypes of stomach cancer defined by site and by histology using the contrast test.^53-55^

We conducted analyses of alcohol intake from specific beverage types (beer, wine, spirits).

Tests evaluating the proportional hazards assumption and linearity of associations were conducted (see Supplementary Section 4). A sensitivity analysis was performed excluding the first 2 years of follow-up to assess reverse causation.

In the cohorts with *H. pylori* infection status, potential confounding by and interaction with *H. pylori* infection status was evaluated using conditional logistic regression models in pooled nested case-control data.

All statistical tests were 2-sided, and *P *< .05 was considered statistically significant. All analyses were conducted using SAS software, version 9.4 (SAS Institute).

## Results

During follow-up (mean 9 to 29 years across 20 cohort studies) of 1 052 025 (52%) women and 957 926 (48%) men, 8357 (2406 women; 5951 men) incident invasive adenocarcinomas of the stomach were diagnosed (Table 1). These included 1867 (22%) cancers of gastric cardia, 4400 (53%) noncardia cancers, and 1832 (22%) overlapping/unspecified tumors, with 258 (3%) uncoded for location. Across studies, 17% to 83% of women and 32% to 93% of men reported consuming any alcohol, with relatively higher median intakes seen for men (10 to 14 g is roughly equivalent to a standard alcoholic beverage serving) (Table 1).

The association between alcohol intake and total stomach cancer incidence was weak overall (Table 2). The age- and sex-adjusted HR comparing ≥30 g/day vs 0.1-<5 g/day (1.16; 95% confidence interval [CI] = 1.05 to 1.28) attenuated markedly when further adjusted for smoking and other confounding factors (multivariable-adjusted HR comparing ≥30 g/day vs 0.1-<5 g/day: 1.06 [95% CI = 0.96 to 1.16]). We observed no material difference by sex (heterogeneity test *P* = .83). There was no evidence of between-studies heterogeneity (for ≥30 g/day category, heterogeneity test *P* = .43 and *I*^2^ statistic = 3%; Figure S1). Among men, owing to their higher alcohol intake, we expanded the highest intake category to 30-<45, 45-<60, and ≥60 g/day; the corresponding HRs were 1.01 (95% CI = 0.87 to 1.16), 1.10 (95% CI = 0.94 to 1.28), and 1.18 (95% CI = 0.97 to 1.44), respectively. In women and men combined, a HR of 1.08 (95% CI = 1.01 to 1.16) was observed for an intake of <0.1 g/day, compared with 0.1-<5 g/day (heterogeneity test *P* = .42 and *I*^2^ statistic = 3%; heterogeneity test *P* value for sex = .91).

**Figure 1.**
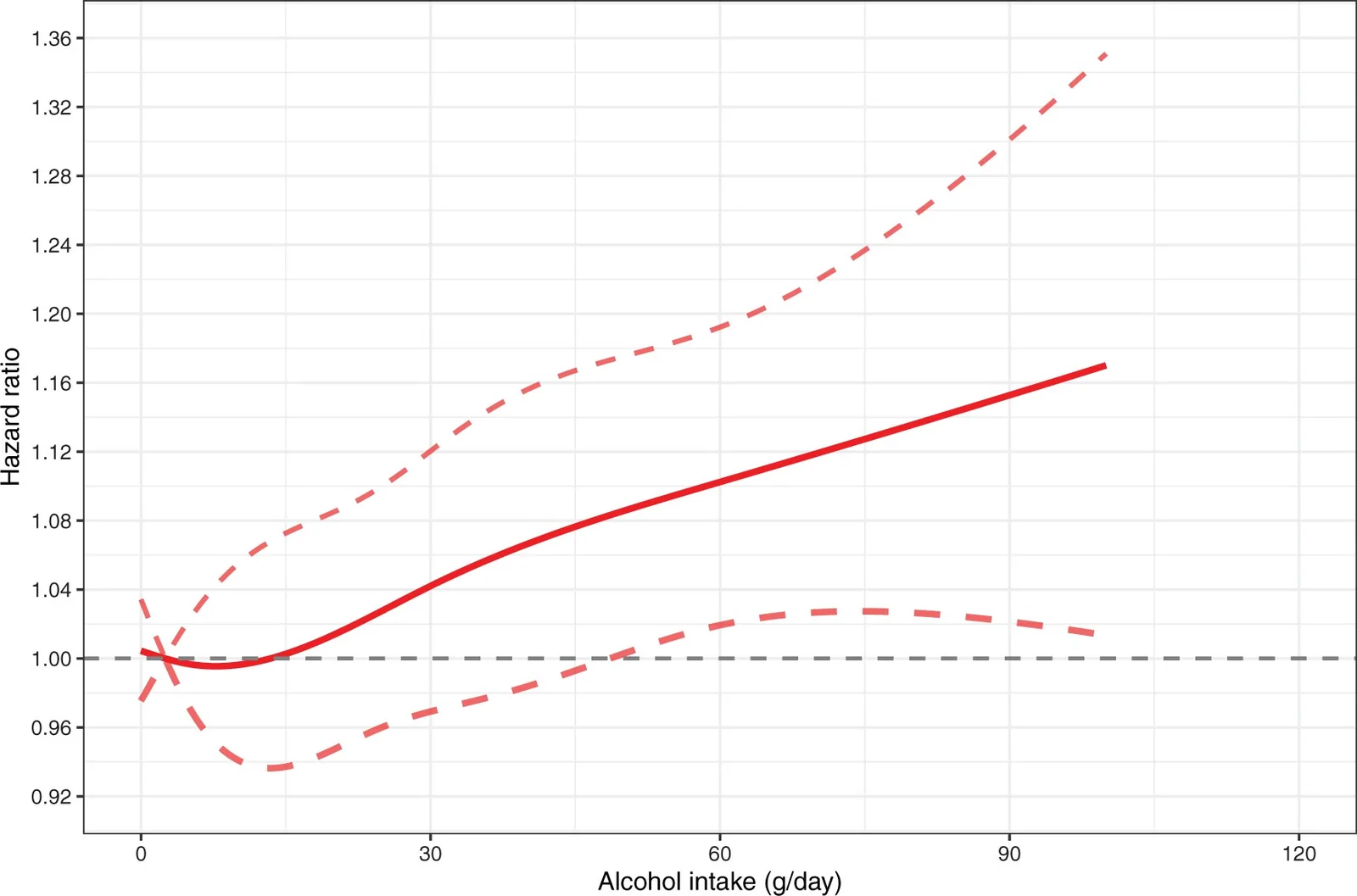
Dose-response spline curve examining relationship between alcohol intake and risk of total stomach cancer. Nonparametric regression curves drawn using restricted cubic spline, with center at 2.5 g/day (the midpoint of reference group 0.1-<5 g/day), with 4 knots placed at 0.1, 5, 30, and 60 g/day and upper intake capped at 100 g/day (Hertzmark E, Li R, Spiegelman D. The SAS MAKESPL Macro: Yale School of Public Health; 2021). Dashed lines represent 95% confidence intervals for spline estimate. *P = .84 for nonlinearity using likelihood ratio test.

We analyzed an aggregated dataset in which the data from all studies were combined that confirmed the findings from the 2-stage analysis for total stomach cancer (HR comparing ≥30 g/day vs 0.1-<5 g/day: 1.07 [95% CI = 0.99 to 1.16]). All remaining analyses were conducted in the aggregated dataset. The model with restricted cubic splines fit no better than a model with a single linear term for alcohol intake (*P* for nonlinearity = .85; Figure 1).

The associations with total stomach cancer incidence differed across smoking strata (*P*_interaction_ = .02), with the strongest positive association for the ≥30 g/day category being observed for never smokers (HR, 1.20 [95% CI = 1.02 to 1.42]) when compared with 0.1-<5 g/day (Table 3). The association with alcohol intake also was stronger in individuals with BMI <25 kg/m^2^ than in those with higher BMI (*P*_interaction_ = .02). However, among never smokers, a stronger association was observed for those with higher BMI (HR, 1.06 per 10 g/day increment [95% CI = 1.02 to 1.09]) than with BMI <25 kg/m^2^ (HR, 1.00 [95% CI = 0.96 to 1.03], *P*_interaction_ = .06). Analyses by geographic region showed the strongest association was in Asian studies (HR comparing ≥30 g/day vs 0.1-<5 g/da: 1.21 [95% CI = 1.02 to 1.42], *P*_interaction_ = .01). There was little evidence of effect modification by age (*P*_interaction_ = .36) and follow-up time (*P*_interaction_ = .20).

**Table 3.** Pooled multivariable hazard ratios (HR)^a^ and 95% confidence intervals (95% CI) for alcohol intake and stomach cancer risk by cigarette smoking status, age, body mass index, follow-up time, and geographic region.

		Alcohol intake categories (g/day)					***P* trend** ^c^	Per 10 g/day increment	***P*, Test for interaction** ^d^
		**Abstention** ^b^	0.1-<5	5-<15	15-<30	≥30			
Smoking status									.02
Never	No. of cases (%)	1548 (53.3)	606 (20.9)	311 (10.7)	223 (7.7)	216 (7.4)		2904 (100)	
	HR (95% CI)	1.06 (0.96 to 1.18)	1.00 (ref)	0.91 (0.79 to 1.04)	1.13 (0.97 to 1.32)	1.20 (1.02 to 1.42)	.01	1.03 (1.00 to 1.05)	
Former	No. of cases (%)	833 (30.5)	638 (23.3)	488 (17.8)	388 (14.2)	389 (14.2)		2736 (100)	
	HR (95% CI)	1.15 (1.03 to 1.28)	1.00 (ref)	0.97 (0.86 to 1.10)	1.03 (0.91 to 1.18)	1.02 (0.89 to 1.17)	.60	1.01 (0.99 to 1.03)	
Current	No. of cases (%)	796 (31.6)	381 (15.1)	358 (14.2)	344 (13.7)	641 (25.4)		2520 (100)	
	HR (95% CI)	0.97 (0.85 to 1.11)	1.00 (ref)	0.99 (0.86 to 1.15)	0.94 (0.81 to 1.09)	1.05 (0.91 to 1.21)	.33	1.01 (0.99 to 1.03)	
Age^e^									.36
<65 years	No. of cases (%)	759 (36.1)	363 (17.2)	317 (15.1)	255 (12.1)	410 (19.5)		2104 (100)	
	HR (95% CI)	1.14 (0.99 to 1.30)	1.00 (ref)	1.05 (0.90 to 1.23)	1.03 (0.87 to 1.22)	1.19 (1.02 to 1.39)	.03	1.02 (1.00 to 1.05)	
65-<75 years	No. of cases (%)	1366 (37.2)	750 (20.4)	529 (14.4)	457 (12.4)	573 (15.6)		3675 (100)	
	HR (95% CI)	1.10 (1.00 to 1.21)	1.00 (ref)	0.95 (0.85 to 1.07)	1.05 (0.93 to 1.19)	1.06 (0.94 to 1.19)	.20	1.01 (0.99 to 1.03)	
≥75 years	No. of cases (%)	1130 (43.8)	556 (21.6)	339 (13.1)	262 (10.2)	291 (11.3)		2578 (100)	
	HR (95% CI)	1.02 (0.91 to 1.13)	1.00 (ref)	0.91 (0.79 to 1.04)	0.98 (0.84 to 1.15)	0.99 (0.84 to 1.15)	.90	1.00 (0.98 to 1.03)	
Body mass index									.02
<25 kg/m^2^	No. of cases (%)	1774 (41.5)	690 (16.1)	562 (13.1)	508 (11.9)	743 (17.4)		4277 (100)	
	HR (95% CI)	1.10 (1.00 to 1.21)	1.00 (ref)	1.00 (0.89 to 1.12)	1.10 (0.98 to 1.24)	1.18 (1.05 to 1.32)	.002	1.02 (1.01 to 1.04)	
≥25 kg/m^2^	No. of cases (%)	1407 (35.9)	940 (24.0)	601 (15.4)	450 (11.5)	517 (13.2)		3915 (100)	
	HR (95% CI)	1.05 (0.96 to 1.15)	1.00 (ref)	0.92 (0.83 to 1.02)	0.95 (0.85 to 1.07)	0.98 (0.87 to 1.10)	>.99	1.00 (0.99 to 1.02)	
Follow-up time									.20
<5 years	No. of cases (%)	759 (37.7)	415 (20.6)	313 (15.5)	243 (12.1)	283 (14.1)		2013 (100)	
	HR (95% CI)	1.15 (1.01 to 1.31)	1.00 (ref)	1.08 (0.93 to 1.25)	1.09 (0.92 to 1.28)	1.05 (0.89 to 1.23)	.70	1.00 (0.98 to 1.02)	
5-<10 years	No. of cases (%)	827 (36.1)	476 (20.8)	341 (14.9)	279 (12.2)	366 (16.0)		2289 (100)	
	HR (95% CI)	1.15 (1.01 to 1.30)	1.00 (ref)	1.01 (0.88 to 1.17)	1.07 (0.92 to 1.25)	1.17 (1.00 to 1.36)	.03	1.02 (1.00 to 1.04)	
≥10 years	No. of cases (%)	1669 (41.2)	778 (19.2)	531 (13.1)	452 (11.1)	625 (15.4)		4055 (100)	
	HR (95% CI)	0.98 (0.89 to 1.08)	1.00 (ref)	0.87 (0.77 to 0.97)	0.94 (0.84 to 1.07)	1.01 (0.90 to 1.13)	.37	1.02 (1.00 to 1.03)	
Geographic region									.01
North America^f^	No. of cases (%)	1387 (40.6)	911 (26.6)	452 (13.2)	342 (10.0)	328 (9.6)		3420 (100)	
	HR (95% CI)	1.14 (1.05 to 1.25)	1.00 (ref)	0.89 (0.80 to 1.00)	1.04 (0.92 to 1.18)	0.97 (0.86 to 1.11)	.94	1.00 (0.99 to 1.03)	
Asia^g^	No. of cases (%)	1483 (51.3)	212 (7.4)	229 (7.9)	298 (10.3)	668 (23.1)		2890 (100)	
	HR (95% CI)	1.00 (0.86 to 1.16)	1.00 (ref)	1.05 (0.87 to 1.27)	1.06 (0.89 to 1.27)	1.21 (1.02 to 1.42)	.01	1.02 (1.01 to 1.03)	
Europe/Australia^h^	No. of cases (%)	385 (18.8)	546 (26.7)	504 (24.6)	334 (16.3)	278 (13.6)		2047 (100)	
	HR (95% CI)	1.06 (0.93 to 1.22)	1.00 (ref)	0.98 (0.87 to 1.11)	0.98 (0.85 to 1.12)	1.01 (0.86 to 1.19)	.87	1.01 (0.98 to 1.04)	

a Sex-specific cohorts were analyzed. Adjusted for race and ethnicity (Asian, Black, Hispanic, White, other), education (<high school, high school, >high school), cigarette smoking status (never, past, current), amount smoked (cigarettes/day, continuous), duration of cigarette smoking (years, continuous), body mass index (kg/m^2^, continuous), red meat intake (g/day, quintiles), fruit intake (g/day, quintiles), and total energy intake (kcal/day, continuous), stratified by age in years, year of questionnaire return, and center (for European Prospective Investigation into Cancer and Nutrition). Analyses for continuous alcohol intake were further adjusted for an indicator variable identifying alcohol drinking status.

b Abstention was defined as <0.1 g/day.

c *P* value, test for trend was calculated in drinkers (intake ≥0.1 g/day) using the Wald test statistic with the median of each intake category fit as a continuous variable.

d *P* value, test for interaction was calculated using the likelihood ratio test for smoking status, body mass index, follow-up time, and geographic region, and using a contrast test for age at diagnosis.

e Age at diagnosis or end of follow-up (attained age).

f North American studies included Canadian National Breast Screening Study, Cancer Prevention Study II Nutrition Cohort, Health Professionals Follow-up Study, Iowa Women’s Health Study, Multiethnic Cohort Study, NIH-AARP Diet and Health Study, Nurses’ Health Study, Prostate, Lung, Colorectal, and Ovarian Cancer Screening Trial, VITamins and Lifestyle (VITAL) Study: Cohort Study of Dietary Supplements and Cancer Risk, and Women’s Health Initiative.

g Asian studies included Japan Public Health Center-based Prospective Study I, Japan Public Health Center-based Prospective Study II, Shanghai Cohort Study, Shanghai Men’s Health Study, and Singapore Chinese Health Study.

h European/Australian studies included Cohort of Swedish Men, European Prospective Investigation into Cancer and Nutrition, Melbourne Collaborative Cohort Study, Netherlands Cohort Study, and Swedish Mammography Cohort.

The HRs for the highest alcohol intake category did not differ materially between cardia and noncardia cancers (*P*_heterogeneity between subtypes_ > .05) (Table 4, Tables S2 and S3). In analyses by anatomical site within the noncardia region, the HRs for an intake of ≥30 g/day, compared with 0.1-<5 g/day, were 1.45 (95% CI = 0.96 to 2.17; *P*_trend_ = .05), 1.32 (95% CI = 1.08 to 1.63; *P*_trend_ = .004), and 1.60 (95% CI = 0.90 to 2.84; *P*_trend_ = .09) for cancers of the fundus, body, and greater curvature, respectively. An overall absence of associations for more distally located pyloric antrum and pylorus was consistently evident when alcohol intake was modeled as a continuous variable (Table 4) and when the analysis was limited to never smokers (Figure S2).

**Table 4.** Pooled multivariable hazard ratios (HR)^a^ and 95% confidence intervals (95% CI) for alcohol intake and risk of stomach cancer subtypes.

		Per 10 g/day increment	***P*, Test for common effects by subtype** ^b^	Alcohol intake categories (g/day)					***P* trend** ^d^	***P*, Test for common effects by subtype, highest category** ^b^
				**Abstention** ^c^	0.1-<5	5-<15	15-<30	≥30		
By anatomical location^g^										
Gastric cardia	No. of cases (%)	1867 (100)	.42^e^	549 (29.5)	454 (24.3)	327 (17.5)	264 (14.1)	273 (14.6)		.73^e^
	HR (95% CI)	1.02 (1.00 to 1.04)		1.11 (0.97 to 1.27)	1.00 (ref)	0.97 (0.84 to 1.12)	1.07 (0.91 to 1.25)	1.05 (0.90 to 1.24)	.38	
Noncardia	No. of cases (%)	4400 (100)		1918 (43.6)	767 (17.4)	533 (12.1)	466 (10.6)	716 (16.3)		
	HR (95% CI)	1.01 (0.99 to 1.02)		1.03 (0.94 to 1.12)	1.00 (ref)	0.97 (0.87 to 1.09)	1.00 (0.89 to 1.13)	1.09 (0.98 to 1.22)	.001	
Fundus	No. of cases (%)	292 (100)		115 (39.4)	48 (16.4)	35 (12.0)	38 (13.0)	56 (19.2)		
	HR (95% CI)	1.07 (1.02 to 1.11)		1.40 (0.98 to 2.00)	1.00 (ref)	1.04 (0.67 to 1.62)	1.41 (0.90 to 2.19)	1.45 (0.96 to 2.17)	.05	
Body	No. of cases (%)	1273 (100)		544 (42.7)	183 (14.4)	149 (11.7)	128 (10.1)	269 (21.1)		
	HR (95% CI)	1.03 (1.00 to 1.05)		1.13 (0.94 to 1.35)	1.00 (ref)	1.07 (0.86 to 1.34)	1.01 (0.80 to 1.28)	1.32 (1.08 to 1.63)	.004	
Greater curvature	No. of cases (%)	153 (100)		54 (35.3)	37 (24.2)	21 (13.7)	16 (10.5)	25 (16.3)		
	HR (95% CI)	1.04 (0.97 to 1.11)		0.71 (0.46 to 1.12)	1.00 (ref)	0.98 (0.57 to 1.69)	1.14 (0.62 to 2.10)	1.60 (0.90 to 2.84)	.09	
Lesser curvature	No. of cases (%)	594 (100)		271 (45.6)	112 (18.9)	78 (13.1)	66 (11.1)	67 (11.3)		
	HR (95% CI)	0.98 (0.93 to 1.03)		1.02 (0.80 to 1.29)	1.00 (ref)	1.01 (0.75 to 1.36)	1.06 (0.77 to 1.46)	0.97 (0.70 to 1.35)	.86	
Pyloric antrum	No. of cases (%)	1814 (100)		841 (46.4)	305 (16.8)	211 (11.6)	191 (10.5)	266 (14.7)		
	HR (95% CI)	0.99 (0.97 to 1.02)		1.01 (0.88 to 1.17)	1.00 (ref)	0.94 (0.79 to 1.13)	0.97 (0.80 to 1.17)	0.97 (0.81 to 1.17)	.92	
Pylorus	No. of cases (%)	274 (100)		93 (34.0)	82 (29.9)	39 (14.2)	27 (9.9)	33 (12.0)		
	HR (95% CI)	0.98 (0.91 to 1.06)		0.72 (0.52 to 1.01)	1.00 (ref)	0.77 (0.52 to 1.14)	0.75 (0.47 to 1.18)	0.70 (0.45 to 1.09)	.16	
By histological subtype^h^										
Diffuse-type	No. of cases (%)	1272 (100)	.26^f^	512 (40.2)	296 (23.3)	183 (14.4)	136 (10.7)	145 (11.4)		.36^f^
	HR (95% CI)	1.00 (0.96 to 1.03)		1.05 (0.90 to 1.23)	1.00 (ref)	0.97 (0.81 to 1.17)	0.98 (0.80 to 1.21)	0.99 (0.80 to 1.23)	.97	
Intestinal-type	No. of cases (%)	6504 (100)		2525 (38.8)	1243 (19.1)	909 (14.0)	775 (11.9)	1052 (16.2)		
	HR (95% CI)	1.02 (1.00 to 1.03)		1.08 (1.01 to 1.17)	1.00 (ref)	0.96 (0.88 to 1.05)	1.05 (0.96 to 1.15)	1.10 (1.01 to 1.21)	.01	

a Sex-specific cohorts were analyzed. Adjusted for race and ethnicity (Asian, Black, Hispanic, White, other), education (<high school, high school, >high school), cigarette smoking status (never, past, current), amount smoked (cigarettes/day, continuous), duration of cigarette smoking (years, continuous), body mass index (kg/m^2^, continuous), red meat intake (g/day, tertiles), fruit intake (g/day, tertiles), and total energy intake (kcal/day, continuous), stratified by age in years, year of questionnaire return, and center (for European Prospective Investigation into Cancer and Nutrition). Analyses for continuous alcohol intake were further adjusted for an indicator variable identifying alcohol drinking status.

b *P* value, test for common effects by subtype was calculated using a contrast test.

c Abstention was defined as <0.1 g/day.

d *P* value, test for trend was calculated in drinkers (intake ≥0.1 g/day) using the Wald test statistic with the median of each intake category fit as a continuous variable.

e Test for common effects: gastric cardia vs noncardia cancers.

f Test for common effects: diffuse-type vs intestinal-type cancers.

g Gastric cardia: International Classification of Diseases (ICD) 9/ICD 10 codes 151.0/C16.0; noncardia: 151.1 to 151.6/C16.1-C16.6; fundus of stomach: 151.3/C16.1; body of stomach: 151.4/C16.2; greater curvature of stomach: 151.6/C16.6; lesser curvature of stomach: 151.5/C16.5; pyloric antrum: 151.2/C16.3; pylorus: 151.1/C16.4; HRs not reported for overlapping/unspecified tumors (*n* = 1832): 151.8/C16.8 and 151.9/C16.9. These analyses do not include the Health Professionals Follow-up Study or Nurses’ Health Study because data on subsite were not available.

h Diffuse-type: International Classification of Diseases for Oncology codes 8141 scirrhous adenocarcinoma; 8142 linitis plastica; 8145 carcinoma, diffuse type; 8490 signet ring cell carcinoma; intestinal-type: 8140 adenocarcinoma, NOS: not otherwise specified; 8143 superficial spreading adenocarcinoma; 8144 adenocarcinoma, intestinal type; 8210 adenocarcinoma in adenomatous polyp; 8211 tubular adenocarcinoma; 8221 adenocarcinoma in multiple adenomatous polyps; 8260 papillary adenocarcinoma; 8261 adenocarcinoma in villous adenoma; 8262 villous adenocarcinoma; 8263 adenocarcinoma in tubulovillous adenoma; HRs not reported for mixed/other/unknown types (*n* = 581): 8010 carcinoma, NOS; 8020 carcinoma, undifferentiated, NOS; 8021 carcinoma, anaplastic, NOS; 8190 trabecular adenocarcinoma; 8201 cribriform carcinoma, NOS; 8213 serrated adenocarcinoma; 8214 parietal cell adenocarcinoma; 8230 solid adenocarcinoma with mucin formation; 8255 adenocarcinoma with mixed subtypes; 8310 clear cell adenocarcinoma; 8320 granular cell adenocarcinoma; 8323 mixed cell adenocarcinoma; 8480 mucinous adenocarcinoma; 8481 mucin-producing adenocarcinoma; 8560 adenosquamous carcinoma; 8574 adenocarcinoma with neuroendocrine differentiation; 8576 hepatoid adenocarcinoma. These analyses do not include the Health Professionals Follow-up Study because data on histology were not available.

The HRs were 0.99 (95% CI = 0.80 to 1.23) for diffuse-type and 1.10 (95% CI = 1.01 to 1.21) for intestinal-type cancers comparing ≥30 g/day vs 0.1-<5 g/day (*P*_heterogeneity between subtypes_ > .05) (Table 4, Tables S2 and Table S3).

In beverage-specific analyses, compared with 0.1-<5 g/day, daily intakes of ≥15 g of alcohol from beer were associated with increased total stomach cancer incidence (HR, 1.20 [95% CI = 1.09 to 1.32]), whereas associations were close to the null for alcohol intake from wine and from spirits (Table 5). Mutually adjusting for alcohol intake from all 3 beverage types in the model did not materially change the estimated effect sizes.

**Table 5.** Pooled multivariable hazard ratios (HR)^a^ and 95% confidence intervals (95% CI) for intakes of alcohol from beer, wine and spirits and stomach cancer risk.

	Alcohol from Beer			Alcohol from Wine			Alcohol from Spirits		
	No. of cases (%)	HR (95% CI)	***P*, Test for common effects by subtype** ^b^	No. of cases (%)	HR (95% CI)	***P*, Test for common effects by subtype** ^b^	No. of cases (%)	HR (95% CI)	***P*, Test for common effects by subtype** ^b^
Total stomach cancer									
Intake categories (g/day)									
Abstention^c^	5081 (60.8)	1.09 (1.03 to 1.16)		5501 (65.8)	1.15 (1.08 to 1.22)		5611 (67.1)	1.10 (1.04 to 1.18)	
0.1-<5	1779 (21.3)	1.00 (ref)		1755 (21.0)	1.00 (ref)		1535 (18.4)	1.00 (ref)	
5-<15	815 (9.7)	1.02 (0.94 to 1.11)		630 (7.5)	0.92 (0.84 to 1.02)		508 (6.1)	1.11 (1.00 to 1.24)	
≥15	682 (8.2)	1.20 (1.09 to 1.32)		471 (5.7)	0.98 (0.88 to 1.09)		703 (8.4)	0.98 (0.89 to 1.08)	
*P* trend^d^		.0001			.59			.57	
Per 10 g/day increment									
All	8357 (100)	1.02 (1.00 to 1.04)		8357 (100)	0.96 (0.93 to 0.99)		8357 (100)	0.99 (0.97 to 1.00)	
Women	2406 (100)	1.00 (0.94 to 1.13)		2406 (100)	0.95 (0.88 to 1.03)		2406 (100)	0.90 (0.83 to 0.97)	
Men	5951 (100)	1.02 (1.00 to 1.04)		5951 (100)	0.96 (0.93 to 1.00)		5951 (100)	0.99 (0.97 to 1.01)	
By anatomical location^g^ (per 10 g/day increment)									
Gastric cardia	1867 (100)	1.01 (0.97 to 1.05)	.13^e^	1867 (100)	0.99 (0.93 to 1.05)	.29^e^	1867 (100)	1.02 (0.99 to 1.05)	.06^e^
Noncardia	3178 (100)	1.03 (1.00 to 1.06)		3178 (100)	0.95 (0.90 to 0.99)		3178 (100)	0.98 (0.96 to 1.00)	
Fundus	292 (100)	0.97 (0.87 to 1.09)		292 (100)	0.93 (0.74 to 1.15)		292 (100)	1.06 (1.00 to 1.13)	
Body	1273 (100)	1.02 (0.97 to 1.07)		1273 (100)	0.97 (0.89 to 1.05)		1273 (100)	0.99 (0.96 to 1.03)	
Greater curvature	153 (100)	1.06 (0.98 to 1.14)		153 (100)	1.10 (0.94 to 1.30)		153 (100)	1.06 (0.97 to 1.16)	
Lesser curvature	594 (100)	1.02 (0.96 to 1.08)		594 (100)	0.99 (0.90 to 1.08)		594 (100)	0.96 (0.89 to 1.03)	
Pyloric antrum	1814 (100)	1.05 (1.01 to 1.09)		1814 (100)	0.89 (0.83 to 0.97)		1814 (100)	0.95 (0.91 to 0.99)	
Pylorus	274 (100)	1.03 (0.91 to 1.16)		274 (100)	1.00 (0.82 to 1.22)		274 (100)	0.96 (0.86 to 1.08)	
By histological subtype^h^ (per 10 g/day increment)									
Diffuse-type	1272 (100)	0.99 (0.94 to 1.04)	.19^f^	1272 (100)	1.01 (0.95 to 1.08)	.12^f^	1272 (100)	0.96 (0.91 to 1.02)	.40^f^
Intestinal-type	6504 (100)	1.03 (1.01 to 1.05)		6504 (100)	0.96 (0.92 to 0.99)		6504 (100)	0.99 (0.97 to 1.00)	

a Sex-specific cohorts were analyzed. Adjusted for race and ethnicity (Asian, Black, Hispanic, White, other), education (<high school, high school, >high school), cigarette smoking status (never, past, current), amount smoked (cigarettes/day, continuous), duration of cigarette smoking (years, continuous), body mass index (kg/m^2^, continuous), red meat intake (g/day, quintiles), fruit intake (g/day, quintiles), and total energy intake (kcal/day, continuous), stratified by age in years, year of questionnaire return, and center (for European Prospective Investigation into Cancer and Nutrition).

b *P* value, test for common effects by subtype for highest category was calculated using a contrast test.

c Abstention was defined as <0.1 g/day.

d *P* value, test for trend was calculated in drinkers (intake ≥0.1 g/day) using the Wald test statistic with the median of each intake category fit as a continuous variable.

e Test for common effects: gastric cardia vs noncardia cancers.

f Test for common effects: diffuse-type vs intestinal-type cancers.

g Gastric cardia: International Classification of Diseases (ICD) 9/ICD 10 codes C16.0; noncardia: 151.1 to 151.6/C16.1-C16.6; fundus of stomach: 151.3/C16.1; body of stomach: 151.4/C16.2; greater curvature of stomach: 151.6/C16.6; lesser curvature of stomach: 151.5/C16.5; pyloric antrum: 151.2/C16.3; pylorus: 151.1/C16.4; HRs not reported for overlapping/unspecified tumors (*n* = 1832): 151.8/C16.8 and 151.9/C16.9. These analyses do not include the Health Professionals Follow-up Study or Nurses’ Health Study because data on subsite were not available.

h Diffuse-type: International Classification of Diseases for Oncology codes 8141 scirrhous adenocarcinoma; 8142 linitis plastica; 8145 carcinoma, diffuse type; 8490 signet ring cell carcinoma; intestinal-type: 8140 adenocarcinoma, NOS: not otherwise specified; 8143 superficial spreading adenocarcinoma; 8144 adenocarcinoma, intestinal type; 8210 adenocarcinoma in adenomatous polyp; 8211 tubular adenocarcinoma; 8221 adenocarcinoma in multiple adenomatous polyps; 8260 papillary adenocarcinoma; 8261 adenocarcinoma in villous adenoma; 8262 villous adenocarcinoma; 8263 adenocarcinoma in tubulovillous adenoma; HRs not reported for mixed/other/unknown types (*n* = 581): 8010 carcinoma, NOS; 8020 carcinoma, undifferentiated, NOS; 8021 carcinoma, anaplastic, NOS; 8190 trabecular adenocarcinoma; 8201 cribriform carcinoma, NOS; 8213 serrated adenocarcinoma; 8214 parietal cell adenocarcinoma; 8230 solid adenocarcinoma with mucin formation; 8255 adenocarcinoma with mixed subtypes; 8310 clear cell adenocarcinoma; 8320 granular cell adenocarcinoma; 8323 mixed cell adenocarcinoma; 8480 mucinous adenocarcinoma; 8481 mucin-producing adenocarcinoma; 8560 adenosquamous carcinoma; 8574 adenocarcinoma with neuroendocrine differentiation; 8576 hepatoid adenocarcinoma. These analyses do not include the Health Professionals Follow-up Study because data on histology were not available.

Among the 6 studies with data on *H. pylori* infection status, the multivariable-adjusted odds ratio (OR) for a 10 g/day increment in alcohol intake with adjustment for *H. pylori* infection status did not differ materially from the one without adjustment for infection status. Overall, there was no strong evidence for an interaction between alcohol intake and *H. pylori* infection status (*P*_interaction_ > .05) (Table S4). The direction of the associations with total stomach and noncardia cancer, however, was not identical between the full cohort (eg, HR for stomach cancer, 1.02 [95% CI = 1.00 to 1.04]) and nested case-control (eg, OR, 0.95 [95% CI = 0.91 to 1.00]) data for these cohorts.

Most associations between alcohol intake and incidence of total stomach cancer and its subtypes did not change appreciably when the first 2 years of follow-up were excluded (712 cases excluded), although the effect sizes for the ≥30 g/day category for fundus, body, and greater curvature were slightly larger than those including all follow-up (Table S5).

## Discussion

We conducted by far the largest pooled prospective analysis to date that assessed associations between alcohol intake and stomach cancer risk and, to our knowledge, examined associations for each specific subsite for the first time. In this pooled analysis, evidence for an association with the risk of total stomach cancer was weak overall. Positive associations were observed, however, for total stomach cancer for never smokers, limiting the likelihood of residual confounding by smoking, and for Asian studies. We observed some evidence of increased risk of proximally located noncardia cancers with increasing alcohol intake.

Findings from individual studies have been inconsistent on the role of alcohol in stomach carcinogenesis, and meta-analyses have supported a potential positive association.^5^^,^^56^^,^^57^ As we also observed, a World Cancer Research Fund/American Institute for Cancer Research (WCRF/AICR) meta-analysis of 21 prospective cohort and 2 nested case-control studies, including 11 926 stomach cancer cases, reported weak positive associations for overall (pooled relative risk [RR] per 10 g/day increment in alcohol intake: 1.02 [95% CI = 1.00 to 1.04]), cardia (RR, 1.01 [95% CI = 0.99 to 1.03]), and noncardia (RR, 1.03 [95% CI = 0.97 to 1.09]) stomach cancer.^5^ Six cohorts overlap between our pooled analysis and that meta-analysis;^13^^,^^15^^,^^16^^,^^19^^,^^20^^,^^25^ additional studies included in the meta-analysis often did not use a validated FFQ and/or defined stomach cancer mortality as the endpoint.^5^ On the other hand, a pooled analysis of 10 hospital-based, 8 population-based, and 2 nested case-control study datasets found increased risks for both cardia (OR, 1.61 [95% CI = 1.11 to 2.34]) and noncardia (OR, 1.28 [95% CI = 1.13 to 1.45]) cancers for intakes above 4 drinks/day compared with nondrinking.^58^ Potential selection bias, especially for studies including hospital controls, may have affected the latter results.

Ethanol in alcoholic beverages is absorbed by the small intestine and later metabolized, mainly in the liver, to acetaldehyde and then to acetate by enzymes alcohol dehydrogenase (ADH) and aldehyde dehydrogenase (ALDH), respectively.^59^ Acetaldehyde is both carcinogenic^60^ and mutagenic.^61^ First-pass metabolism of ethanol, predominantly in the gastric mucosa in the body of the stomach,^62^ also produces acetaldehyde^63^ following high alcohol concentrations when the stomach is full,^64^ which possibly explains higher risks observed in the present study for several proximally located noncardia subsites. Other potential mechanisms explaining the carcinogenic effect of alcohol include via interference with retinoid metabolism leading to adverse effects on cellular differentiation and apoptosis, the production of lipid peroxidation and oxygen free radicals, or by direct cellular injury and gene mutation by enhancing penetration of environmental carcinogens (eg, tobacco) into cells.^5^ The role of ethanol metabolism in tumorigenesis is related to the polymorphisms in genes involved in the oxidation of ethanol.^6^^,^^9^ We observed a stronger association between alcohol intake and stomach cancer risk in Asian studies compared with studies conducted in Australia, Europe, and North America. This suggestive positive finding in our pooled analyses is in line with the findings of a prior pooled analysis of 6 cohorts (2 are included here^16^) conducted in Japan, which reported positive associations for intakes of ≥23 g/day, compared with abstention, for regular drinking in men; the HR for women for the corresponding intake was 1.22 (0.98-1.53).^65^ The same study reported relatively stronger associations for heavy-drinking females (≥23 g/day) and males (≥92 g/day) for proximal stomach cancer than for distal cancer,^65^ consistent with our findings. One possible explanation for the positive association observed in Asian populations may be due to their high prevalence of the genotype ALDH2*2, leading to excessive accumulation of acetaldehyde.^66-68^

Our study had several strengths. We harmonized and pooled participant-level data from 20 cohort studies including more than 1.8 million individuals and more than 8300 incident stomach cancers, to our knowledge the largest investigation using pooled prospective data of the association between alcohol intake and stomach cancer risk. We included studies across diverse populations from 4 continents, and we examined associations, often with high precision, with each ICD-defined subsite within the stomach, hitherto unreported, even in large consortia projects.^5^^,^^58^ The studies were also selected into a consortium evaluating risk factors for stomach cancer more broadly and included 10 studies that collectively contributed 2412 cases that have not published data on alcohol and stomach cancer;^10-12^^,^^14^^,^^15^^,^^18^^,^^21^^,^^26^^,^^27^ hence we minimized publication bias.

The present study also had several potential limitations. We cannot completely rule out misclassification of stomach cancers by subtype, because 25% were classified as overlapping/unspecified or missing for subsite, a well-recognized difficulty in verifying the origin of stomach cancers.^69^ Another limitation is that individuals who give up drinking due to poor health (sometimes referred to as “sick quitters”) are usually classified as nondrinkers,^70^ and individuals with lifelong illnesses that could affect stomach cancer risk may never drink alcohol,^71^ possibly leading to the increased risk we observed for an intake of <0.1 g/day. The use of the intake category 0.1-<5 g/day as the reference category minimized the overall impact of this phenomenon. We aimed to address the “sick quitter” hypothesis further in a sensitivity analysis (Supplementary Section 5; Table S6). Although misclassification of self-reported alcohol intake cannot be ruled out completely, intakes reported by cohort participants have consistently shown high validity. Although we observed evidence of heterogeneity across some subgroups and subtypes, these findings should be interpreted with caution unless supported by prior hypotheses or replicated in other studies because multiple tests were performed, increasing the likelihood of chance findings, in addition to misclassification of subtypes. For example, we also observed a positive association for alcohol from beer, whereas associations for wine and spirits were closer to null. This pattern should be interpreted cautiously and presently lacks a plausible biological mechanism. Although beverage-specific associations may reflect differences in drinking behaviors or regional and cultural differences in beverage preference, this may also represent a chance finding and requires replication. Residual confounding (eg, due to smoking, despite adjusting for 3 measures of smoking habits) is possible. Yet, the strongest positive association with alcohol consumption was observed in never smokers. In contrast, the stronger association observed for participants with normal weight did not persist when the analysis was limited to never smokers. Lastly, although we found no evidence of confounding or effect modification by *H. pylori* infection status using nested case-control data from 6 cohort studies,^13^^,^^16^^,^^17^^,^^21^^,^^23^ nested case-control data may not be fully representative of the entire cohort and the risk estimates vary. The association observed in the nested case-control analysis differed from that seen in the full cohort analysis of these 6 cohorts, but these findings are not directly comparable. In addition, we were unable to account for regional variation in *H. pylori* strain virulence or related bacterial factors because harmonized data on these characteristics were not available across studies.

In summary, in this pooled analysis of individual-level data from 20 cohorts, higher alcohol intake was not associated with increased risk of overall stomach cancer; however, we observed suggestive evidence of associations in Asian studies, as has been observed previously, and in never smokers. The potential mechanisms that may explain alcohol-induced gastric carcinogenesis merit additional research.

## Supplementary Material

pkag073_Supplementary_Data

## Data Availability

The sharing of data will be coordinated by the corresponding author and governed by executed data use agreements between the home institution of the cohort study and the Harvard T.H. Chan School of Public Health, as well as approval from lead investigators or the leadership group for each cohort.

## References

[1] Bray F , Laversanne M, Sung H, et al Global cancer statistics 2022: GLOBOCAN estimates of incidence and mortality worldwide for 36 cancers in 185 countries. CA Cancer J Clin. 2024;74:229-263. 10.3322/caac.21834 38572751

[2] Thrift AP , El-Serag HB. Burden of gastric cancer. Clin Gastroenterol Hepatol. 2020;18:534-542. 10.1016/j.cgh.2019.07.045 PMC8859863 31362118

[3] Bass AJ , Thorsson V, Shmulevich I, et al Comprehensive molecular characterization of gastric adenocarcinoma. Nature. 2014;513:202-209. 10.1038/nature13480 PMC4170219 25079317

[4] Amin MB , Edge S, Greene F, et al AJCC Cancer Staging Manual (8th edition). Springer International Publishing, American Joint Commission on Cancer; 2017.

[5] World Cancer Research Fund International/American Institute for Cancer Research. Continuous Update Project Report: Diet, Nutrition, Physical Activity and Stomach Cancer (available at: wcrf.org/stomach-cancer-2016); 2016.

[6] International Agency for Research on Cancer Working Group on the Evaluation of Carcinogenic Risks to Humans. IARC Monographs on the Evaluation of Carcinogenic Risks to Humans, vol 96 Alcohol Consumption and Ethyl Carbamate. Lyon, France, International Agency for Research on Cancer; 2010. PMC4781168 21735939

[7] Baan R , Straif K, Grosse Y, et al; WHO International Agency for Research on Cancer Monograph Working Group. Carcinogenicity of alcoholic beverages. Lancet Oncol. 2007;8:292-293. 10.1016/s1470-2045(07)70099-2 17431955

[8] Office of the Surgeon General. Alcohol and Cancer Risk: The U.S. Surgeon General’s Advisory. US Department of Health and Human Services; 2025. 40367254

[9] LoConte NK , Brewster AM, Kaur JS, et al Alcohol and cancer: a statement of the American Society of Clinical Oncology. J Clin Oncol. 2018;36:83-93. 10.1200/JCO.2017.76.1155 29112463

[10] Miller AB , Wall C, Baines CJ, et al Twenty five year follow-up for breast cancer incidence and mortality of the Canadian National Breast Screening Study: randomised screening trial. BMJ. 2014;348:g366. 10.1136/bmj.g366 PMC3921437 24519768

[11] Calle EE , Rodriguez C, Jacobs EJ, et al The American Cancer Society Cancer Prevention Study II Nutrition Cohort: rationale, study design, and baseline characteristics. Cancer. 2002;94:2490-2501. 10.1002/cncr.101970 12015775

[12] Larsson SC , Bergkvist L, Wolk A. Fruit and vegetable consumption and incidence of gastric cancer: a prospective study. Cancer Epidemiol Biomarkers Prev. 2006;15:1998-2001. 10.1158/1055-9965.EPI-06-0402 17035412

[13] Duell EJ , Travier N, Lujan-Barroso L, et al Alcohol consumption and gastric cancer risk in the European Prospective Investigation into Cancer and Nutrition (EPIC) cohort. Am J Clin Nutr. 2011;94:1266-1275. 10.3945/ajcn.111.012351 21993435

[14] Kwon S , Ma W, Drew DA, et al Association between aspirin use and gastric adenocarcinoma: a prospective cohort study. Cancer Prev Res (Phila). 2022;15:265-272. 10.1158/1940-6207.CAPR-21-0413 PMC10022803 34980677

[15] Zheng W , Sellers TA, Doyle TJ, et al Retinol, antioxidant vitamins, and cancers of the upper digestive tract in a prospective cohort study of postmenopausal women. Am J Epidemiol. 1995;142:955-960. 10.1093/oxfordjournals.aje.a117743 7572976

[16] Sasazuki S , Sasaki S, Tsugane S; Japan Public Health Center Study Group. Cigarette smoking, alcohol consumption and subsequent gastric cancer risk by subsite and histologic type. Int J Cancer. 2002;101:560-566. 10.1002/ijc.10649 12237898

[17] Jayasekara H , MacInnis RJ, Lujan-Barroso L, et al Lifetime alcohol intake, drinking patterns over time and risk of stomach cancer: a pooled analysis of data from two prospective cohort studies. Int J Cancer. 2021;148:2759-2773. 10.1002/ijc.33504 PMC9290950 33554339

[18] Nomura AM , Wilkens LR, Henderson BE, et al The association of cigarette smoking with gastric cancer: the multiethnic cohort study. Cancer Causes Control. 2012;23:51-58. 10.1007/s10552-011-9854-0 PMC4166441 22037905

[19] Steevens J , Schouten LJ, Goldbohm RA, et al Alcohol consumption, cigarette smoking and risk of subtypes of oesophageal and gastric cancer: a prospective cohort study. Gut. 2010;59:39-48. 10.1136/gut.2009.191080 19828467

[20] Wang S , Freedman ND, Loftfield E, et al Alcohol consumption and risk of gastric cardia adenocarcinoma and gastric noncardia adenocarcinoma: a 16-year prospective analysis from the NIH-AARP diet and health cohort. Int J Cancer. 2018;143:2749-2757. 10.1002/ijc.31740 PMC7579679 29992560

[21] In H , Sarkar S, Ward J, et al Serum pepsinogen as a biomarker for gastric cancer in the United States: a nested case-control study using the PLCO cancer screening trial data. Cancer Epidemiol Biomarkers Prev. 2022;31:1426-1432. 10.1158/1055-9965.EPI-21-1328 PMC9268394 35534235

[22] Moy KA , Fan Y, Wang R, et al Alcohol and tobacco use in relation to gastric cancer: a prospective study of men in Shanghai, China. Cancer Epidemiol Biomarkers Prev. 2010;19:2287-2297. 10.1158/1055-9965.EPI-10-0362 PMC2936659 20699372

[23] Epplein M , Shu XO, Xiang YB, et al Fruit and vegetable consumption and risk of distal gastric cancer in the Shanghai Women’s and Men’s Health studies. Am J Epidemiol. 2010;172:397-406. 10.1093/aje/kwq144 PMC2950795 20647333

[24] Wang Z , Koh WP, Jin A, et al Composite protective lifestyle factors and risk of developing gastric adenocarcinoma: the Singapore Chinese Health Study. Br J Cancer. 2017;116:679-687. 10.1038/bjc.2017.7 PMC5344300 28125822

[25] Larsson SC , Giovannucci E, Wolk A. Alcoholic beverage consumption and gastric cancer risk: a prospective population-based study in women. Int J Cancer. 2007;120:373-377. 10.1002/ijc.22204 17066442

[26] White E , Patterson RE, Kristal AR, et al VITamins and Lifestyle cohort study: study design and characteristics of supplement users. Am J Epidemiol. 2004;159:83-93. 10.1093/aje/kwh010 14693663

[27] Brasky TM , Ray RM, Newton AM, et al Supplemental B-vitamins and risk of upper gastrointestinal cancers in the Women’s Health Initiative. Nutr Cancer. 2023;75:1103-1108. 10.1080/01635581.2023.2186258 36895169

[28] Smith-Warner SA , Spiegelman D, Ritz J, et al Methods for pooling results of epidemiologic studies: the pooling project of prospective studies of diet and cancer. Am J Epidemiol. 2006;163:1053-1064. 10.1093/aje/kwj127 16624970

[29] Swerdlow AJ , Harvey CE, Milne RL, et al The National Cancer Institute Cohort Consortium: an international pooling collaboration of 58 cohorts from 20 countries. Cancer Epidemiol Biomarkers Prev. 2018;27:1307-1319. 10.1158/1055-9965.EPI-18-0182 30018149

[30] Flagg EW , Coates RJ, Calle EE, et al Validation of the American Cancer Society Cancer Prevention Study II nutrition survey cohort food frequency questionnaire. Epidemiology. 2000;11:462-468. 10.1097/00001648-200007000-00017 10874556

[31] Kaaks R , Slimani N, Riboli E: Pilot phase studies on the accuracy of dietary intake measurements in the EPIC project: overall evaluation of results. European Prospective Investigation into Cancer and Nutrition. Int J Epidemiol 1997;26 Suppl 1:S26-36. 10.1093/ije/26.suppl_1.s26 9126531

[32] Giovannucci E , Colditz G, Stampfer MJ, et al The assessment of alcohol consumption by a simple self-administered questionnaire. Am J Epidemiol. 1991;133:810-817. 10.1093/oxfordjournals.aje.a115960 2021148

[33] Munger RG , Folsom AR, Kushi LH, et al Dietary assessment of older Iowa women with a food frequency questionnaire: nutrient intake, reproducibility, and comparison with 24-hour dietary recall interviews. Am J Epidemiol. 1992;136:192-200. 10.1093/oxfordjournals.aje.a116485 1415141

[34] Tsubono Y , Kobayashi M, Sasaki S, et al; JPHC. Validity and reproducibility of a self-administered food frequency questionnaire used in the baseline survey of the JPHC Study Cohort I. J Epidemiol. 2003;13:S125-33. 10.2188/jea.13.1sup_125 PMC9767689 12701640

[35] Ishihara J , Sobue T, Yamamoto S, et al; JPHC. Validity and reproducibility of a self-administered food frequency questionnaire in the JPHC Study Cohort II: study design, participant profile and results in comparison with Cohort I. J Epidemiol. 2003;13:S134-147. 10.2188/jea.13.1sup_134 PMC9767691 12701641

[36] Park SY , Wilkens LR, Setiawan VW, et al Alcohol intake and colorectal cancer risk in the multiethnic cohort study. Am J Epidemiol. 2019;188:67-76. 10.1093/aje/kwy208 PMC6321802 30239578

[37] Goldbohm RA , van den Brandt PA, Brants HA, et al Validation of a dietary questionnaire used in a large-scale prospective cohort study on diet and cancer. Eur J Clin Nutr. 1994;48:253-265. 8039485

[38] Millen AE , Midthune D, Thompson FE, et al The National Cancer Institute diet history questionnaire: validation of pyramid food servings. Am J Epidemiol 2006;163:279-288. 10.1093/aje/kwj031 16339051

[39] Villegas R , Yang G, Liu D, et al Validity and reproducibility of the food-frequency questionnaire used in the Shanghai Men’s Health Study. Br J Nutr. 2007;97:993-1000. 10.1017/S0007114507669189 17381986

[40] Hankin JH , Stram DO, Arakawa K, et al Singapore Chinese Health Study: development, validation, and calibration of the quantitative food frequency questionnaire. Nutr Cancer. 2001;39:187-195. 10.1207/S15327914nc392_5 11759279

[41] Khani BR , Ye W, Terry P, et al Reproducibility and validity of major dietary patterns among Swedish women assessed with a food-frequency questionnaire. J Nutr. 2004;134:1541-1545. 10.1093/jn/134.6.1541 15173426

[42] Patterson RE , Kristal AR, Tinker LF, et al Measurement characteristics of the Women’s Health Initiative food frequency questionnaire. Ann Epidemiol. 1999;9:178-187. 10.1016/s1047-2797(98)00055-6 10192650

[43] Puckett C. The Educational Annotation of ICD-9-CM; Diseases and Procedures Tabular Lists. 1986.

[44] World Health Organization. International statistical classification of diseases and related health problems, 10th revision. Geneva, World Health Organization; 2011.

[45] Owen DA. Stomach. In: Mills SE, ed. Histology for Pathologists. 4th ed. Wolters Kluwer Health; 2012:633-646.

[46] World Health Organization. International classification of diseases for oncology (ICD-O). 3rd edition, 1st revision. Geneva, World Health Organization; 2013.

[47] Prentice RL. A case-cohort design for epidemiologic cohort studies and disease prevention trials. Biometrika. 1986;73:1-11. 10.1093/biomet/73.1.1

[48] Korn EL , Graubard BI, Midthune D. Time-to-event analysis of longitudinal follow-up of a survey: choice of the time-scale. Am J Epidemiol. 1997;145:72-80. 10.1093/oxfordjournals.aje.a009034 8982025

[49] DerSimonian R , Laird N. Meta-analysis in clinical trials. Control Clin Trials. 1986;7:177-188. 10.1016/0197-2456(86)90046-2 3802833

[50] Higgins JPT , Thompson SG, Deeks JJ, et al Measuring inconsistency in meta-analyses. BMJ. 2003;327:557-560. 10.1136/bmj.327.7414.557 PMC192859 12958120

[51] Stram DO. Meta-analysis of published data using a linear mixed-effects model. Biometrics. 1996;52:536-544. 10.2307/2532893 8672702

[52] Ioannidis JP , Rosenberg PS, Goedert JJ, et al; International Meta-Analysis of HIV Host Genetics. Commentary: Meta-analysis of individual participants’ data in genetic epidemiology. Am J Epidemiol. 2002;156:204-210. 10.1093/aje/kwf031 12142254

[53] Prentice RL , Breslow NE. Retrospective studies and failure time models. Biometrika. 1978;65:153-158. 10.1093/biomet/65.1.153

[54] Anderson TW. An Introduction to Multivariate Statistical Analysis. Wiley-Interscience; 2003.

[55] Wang M , Spiegelman D, Kuchiba A, et al Statistical methods for studying disease subtype heterogeneity. Stat Med. 2016;35:782-800. 10.1002/sim.6793 PMC4728021 26619806

[56] Tramacere I , Negri E, Pelucchi C, et al A meta-analysis on alcohol drinking and gastric cancer risk. Ann Oncol. 2012;23:28-36. 10.1093/annonc/mdr135 21536659

[57] Fang X , Wei J, He X, et al Landscape of dietary factors associated with risk of gastric cancer: a systematic review and dose-response meta-analysis of prospective cohort studies. Eur J Cancer. 2015;51:2820-2832. 10.1016/j.ejca.2015.09.010 26589974

[58] Rota M , Pelucchi C, Bertuccio P, et al Alcohol consumption and gastric cancer risk: a pooled analysis within the StoP project consortium. Int J Cancer. 2017;141:1950-1962. 10.1002/ijc.30891 28718913

[59] Druesne-Pecollo N , Tehard B, Mallet Y, et al Alcohol and genetic polymorphisms: effect on risk of alcohol-related cancer. Lancet Oncol. 2009;10:173-180. 10.1016/S1470-2045(09)70019-1 19185835

[60] IARC Working Group on the Evaluation of Carcinogenic Risks to Humans. IARC Monographs on the Evaluation of Carcinogenic Risks to Humans. Vol 96, Alcohol Consumption and Ethyl Carbamate. Lyon, France: International Agency for Research on Cancer, 2010. PMC4781168 21735939

[61] Poschl G , Seitz HK. Alcohol and cancer. Alcohol. 2004;39:155-165. 10.1093/alcalc/agh057 15082451

[62] Jelski W , Chrostek L, Szmitkowski M, et al Activity of class I, II, III, and IV alcohol dehydrogenase isoenzymes in human gastric mucosa. Dig Dis Sci. 2002;47:1554-1557. 10.1023/a : 1015871219922 10.1023/a:1015871219922 12141816

[63] Lee S-L , Chau G-Y, Yao C-T, et al Functional assessment of human alcohol dehydrogenase family in ethanol metabolism: significance of first-pass metabolism. Alcohol Clin Exp Res. 2006;30:1132-1142. 10.1111/j.1530-0277.2006.00139.x 16792560

[64] Cederbaum AI. Alcohol metabolism. Clin Liver Dis. 2012;16:667-685. 10.1016/j.cld.2012.08.002 PMC3484320 23101976

[65] Tamura T , Wakai K, Lin Y, et al; Research Group for the Development and Evaluation of Cancer Prevention Strategies in Japan. Alcohol intake and stomach cancer risk in Japan: a pooled analysis of six cohort studies. Cancer Sci. 2022;113:261-276. 10.1111/cas.15172 PMC8748227 34689390

[66] Brennan P , Lewis S, Hashibe M, et al Pooled analysis of alcohol dehydrogenase genotypes and head and neck cancer: a HuGE review. Am J Epidemiol. 2004;159:1-16. 10.1093/aje/kwh003 14693654

[67] Matsuo K , Oze I, Hosono S, et al The aldehyde dehydrogenase 2 (ALDH2) Glu504Lys polymorphism interacts with alcohol drinking in the risk of stomach cancer. Carcinogenesis. 2013;34:1510-1515. 10.1093/carcin/bgt080 23455379

[68] Shin CM , Kim N, Cho SI, et al Association between alcohol intake and risk for gastric cancer with regard to ALDH2 genotype in the Korean population. Int J Epidemiol. 2011;40:1047-1055. 10.1093/ije/dyr067 21507992

[69] T de Martel C , Parsonnet J. Stomach Cancer. In: Thun M, Linet MS, Cerhan JR, et al, eds. Cancer Epidemiol Prevention. 4th ed. Oxford University Press; 2017:593-610.

[70] Fillmore KM , Stockwell T, Chikritzhs T, et al Moderate alcohol use and reduced mortality risk: systematic error in prospective studies and new hypotheses. Ann Epidemiol. 2007;17:S16-23. 10.1016/j.annepidem.2007.01.005 17478320

[71] Ng Fat L , Cable N, Marmot MG, et al Persistent long-standing illness and non-drinking over time, implications for the use of lifetime abstainers as a control group. J Epidemiol Community Health. 2014;68:71-77. 10.1136/jech-2013-202576 24166583

