# Supplementary material for: Alcohol intake and risk of stomach cancer: a pooled analysis of 20 cohorts
Source: JNCI Cancer Spectr. 2026 Jul 24;10(5):pkag073. doi: 10.1093/jncics/pkag073 (PMC13589573; doi:10.1093/jncics/pkag073)
Supplement: Supplementary file 1 [file pkag073_supplementary_data.zip › Supplementary Sections_alcohol_stomach_cancer.docx]

Supplementary Sections

**Section 1. Inclusion/exclusion criteria for cohorts**

Each study except the Shanghai Cohort Study^1^ met the following inclusion criteria: (i) ≥1 publication on any diet and cancer association; (ii) use of a long-term diet assessment that was comprehensive enough to estimate total energy intake; (iii) validation of the dietary assessment tool or a closely related instrument; (iv) assessment of alcohol intake; (v) >10% alcohol drinkers for men or women; and (vi) ascertainment of ≥50 incident cases of adenocarcinoma of the stomach for men or women. The Shanghai Cohort Study^1^ was additionally included in this project because they had assessed alcohol intake, even though they did not meet inclusion criteria (ii) and (iii). For the Prostate, Lung, Colorectal, and Ovarian Cancer Screening Trial^2^ and the VITamins and Lifestyle (VITAL) Study^3^, only male participants were included as there were fewer than 50 cases of stomach cancer for women in each cohort; since <10% of the female participants in the Singapore Chinese Health Study^4^ were alcohol drinkers, only males were included.

**Section 2. Assessment of dietary and non-dietary factors**

All studies had information on age, sex, height, weight, and smoking habits. Body mass index (BMI) was calculated as weight in kilograms divided by the square of height in meters (kg/m^2^). Most studies had information on race or ethnicity and education.^5^ Intakes of red meat and fruit, were calculated by summing the intakes of specific foods included in those groups.

**Section 3. *Helicobacter pylori* serostatus**

In six cohorts,^2 6-9^ *H. pylori* serostatus was determined in nested case-control studies on stomach cancer by using a combination of ELISA and immunoblotting^10^ assays in serum or plasma. Details on establishing *H. pylori*-seropositivity for each study have been published previously.^2 11-15^ We used *H. pylori* status as recommended by each study based on their testing protocol. A combination of the following factors was used to match cases and controls in individual studies: age and sex^2 6-9^, race and ethnicity^21^, country of birth or study center^6-8^, date of blood collection^6 7 9^, fasting time at blood collection^7^, and antibiotic use in the week prior to blood collection.^9^

**Section 4. Statistical analysis**

Participants were excluded from the analyses if they met the exclusion criteria specified within each study, were diagnosed with cancer (except non-melanoma skin cancer) prior to baseline, reported energy intakes beyond three standard deviations from their study-specific log_e_-transformed mean energy intake, had missing data on alcohol intake, or reported alcohol intakes of >200 g/day.

We evaluated the main exposures and covariates for divergence from the proportional hazards assumption using a test based on Schoenfeld residuals^16^ and did not find evidence of violation (*P*>0.05).

To test for nonlinearity in associations with alcohol intake, we compared nonparametric regression curves using restricted cubic splines (with 4 knots placed at 0.1, 5, 30 and 60 g/day and upper intake capped at 100 g/day) with the linear model using the likelihood ratio test.^17 18^

**Section 5. Associations in cohorts assessing former drinking**

We aimed to address the ‘*sick quitter’ hypothesis* further in a sensitivity analysis by refining the risk estimate for former drinking in cohorts that assessed former drinking^2 6 8 19-21^ where lifetime abstention was separated out. This additional analysis, however, lacked the cohorts^22-24^ that were primarily responsible for the increased stomach cancer risk observed for an intake of <0.1 g/day. The findings are given in Supplementary Table 6.

**References for supplementary sections**

1. Moy KA, Fan Y, Wang R, et al. Alcohol and tobacco use in relation to gastric cancer: a prospective study of men in Shanghai, China. *Cancer Epidemiol Biomarkers Prev* 2010;19(9):2287-97. doi: 10.1158/1055-9965.EPI-10-0362 [published Online First: 2010/08/12]

2. In H, Sarkar S, Ward J, et al. Serum pepsinogen as a biomarker for gastric cancer in the United States: A nested case-control study using the PLCO cancer screening trial data. *Cancer Epidemiol Biomarkers Prev* 2022;31(7):1426-32.

3. White E, Patterson RE, Kristal AR, et al. VITamins And Lifestyle cohort study: study design and characteristics of supplement users. *Am J Epidemiol* 2004;159(1):83-93. doi: 10.1093/aje/kwh010 [published Online First: 2003/12/25]

4. Wang Z, Koh WP, Jin A, et al. Composite protective lifestyle factors and risk of developing gastric adenocarcinoma: the Singapore Chinese Health Study. *Br J Cancer* 2017;116(5):679-87. doi: 10.1038/bjc.2017.7 [published Online First: 2017/01/27]

5. Smith-Warner SA, Spiegelman D, Ritz J, et al. Methods for pooling results of epidemiologic studies: the Pooling Project of Prospective Studies of Diet and Cancer. *Am J Epidemiol* 2006;163(11):1053-64. doi: doi: 10.1093/aje/kwj127 [published Online First: 2006 Apr 19]

6. Duell EJ, Travier N, Lujan-Barroso L, et al. Alcohol consumption and gastric cancer risk in the European Prospective Investigation into Cancer and Nutrition (EPIC) cohort. *Am J Clin Nutr* 2011;94(5):1266-75. doi: 10.3945/ajcn.111.012351 [published Online First: 2011/10/14]

7. Sasazuki S, Sasaki S, Tsugane S, et al. Cigarette smoking, alcohol consumption and subsequent gastric cancer risk by subsite and histologic type. *Int J Cancer* 2002;101(6):560-66. doi: 10.1002/ijc.10649 [published Online First: 2002/09/19]

8. Jayasekara H, MacInnis RJ, Lujan-Barroso L, et al. Lifetime alcohol intake, drinking patterns over time and risk of stomach cancer: A pooled analysis of data from two prospective cohort studies. *Int J Cancer* 2021;148(11):2759-73. doi: 10.1002/ijc.33504 [published Online First: 2021/02/09]

9. Epplein M, Shu XO, Xiang YB, et al. Fruit and vegetable consumption and risk of distal gastric cancer in the Shanghai Women's and Men's Health studies. *Am J Epidemiol* 2010;172(4):397-406.

10. Monteiro L, de Mascarel A, Sarrasqueta AM, et al. Diagnosis of Helicobacter pylori infection: Noninvasive methods compared to invasive methods and evaluation of two new tests. *Am J Gastroenterol* 2001;96(2):353-58.

11. González CA, Megraud F, Buissonniere A, et al. Helicobacter pylori infection assessed by ELISA and by immunoblot and noncardia gastric cancer risk in a prospective study: the Eurgast-EPIC project. *Ann Oncol* 2012;23(5):1320-24. doi: 10.1093/annonc/mdr384

12. Hamashima C, Sasazuki S, Inoue M, et al. Receiver operating characteristic analysis of prediction for gastric cancer development using serum pepsinogen and Helicobacter pylori antibody tests. *BMC Cancer* 2017;17(1):183.

13. Charvat H, Sasazuki S, Inoue M, et al. Prediction of the 10-year probability of gastric cancer occurrence in the Japanese population: the JPHC study cohort II. *Int J Cancer* 2016;138(2):320-31.

14. Dugue PA, Bassett JK, Brinkman MT, et al. Dietary intake of nutrients involved in one-carbon metabolism and risk of gastric cancer: a prospective study. *Nutrition and Cancer* 2019;71(4):605-14. doi: 10.1080/01635581.2019.1577982

15. Epplein M, Zheng W, Xiang YB, et al. Prospective study of Helicobacter pylori biomarkers for gastric cancer risk among Chinese men. *Cancer Epidemiol Biomarkers Prev* 2012;21(12):2185-92.

16. Grambsch PM, Therneau TM. Proportional hazards tests and diagnostics based on weighted residuals. *Biometrika* 1994;81:515–26.

17. Durrleman S, Simon R. Flexible regression models with cubic splines. *Stat Med* 1989;8:551–61.

18. Harrell FE Jr, Lee KL, Pollock BG. Regression models in clinical studies: determining relationships between predictors and response. *J Natl Cancer Inst* 1988;80(15):1198-202.

19. Larsson SC, Bergkvist L, Wolk A. Fruit and vegetable consumption and incidence of gastric cancer: a prospective study. *Cancer Epidemiol Biomarkers Prev* 2006;15(10):1998-2001. doi: 10.1158/1055-9965.EPI-06-0402 [published Online First: 2006/10/13]

20. Kwon S, Ma W, Drew DA, et al. Association between aspirin use and gastric adenocarcinoma: A prospective cohort study. *Cancer Prev Res (Phila)* 2022;15(4):265-72.

21. Larsson SC, Giovannucci E, Wolk A. Alcoholic beverage consumption and gastric cancer risk: a prospective population-based study in women. *Int J Cancer* 2007;120(2):373-77. doi: 10.1002/ijc.22204 [published Online First: 2006/10/27]

22. Nomura AM, Wilkens LR, Henderson BE, et al. The association of cigarette smoking with gastric cancer: the Multiethnic Cohort Study. *Cancer Causes Control* 2012;23(1):51-58. doi: 10.1007/s10552-011-9854-0 [published Online First: 2011/11/01]

23. Wang S, Freedman ND, Loftfield E, et al. Alcohol consumption and risk of gastric cardia adenocarcinoma and gastric noncardia adenocarcinoma: A 16-year prospective analysis from the NIH-AARP diet and health cohort. *Int J Cancer* 2018;143(11):2749-57. doi: 10.1002/ijc.31740 [published Online First: 2018/07/12]

24. Calle EE, Rodriguez C, Jacobs EJ, et al. The American Cancer Society Cancer Prevention Study II Nutrition Cohort: rationale, study design, and baseline characteristics. *Cancer* 2002;94(9):2490-501.
