# Supplementary material for: Alcohol intake and risk of stomach cancer: a pooled analysis of 20 cohorts
Source: JNCI Cancer Spectr. 2026 Jul 24;10(5):pkag073. doi: 10.1093/jncics/pkag073 (PMC13589573; doi:10.1093/jncics/pkag073)
Supplement: Supplementary file 1 [file pkag073_supplementary_data.zip › Supplemental Data_alcohol_stomach cancer.docx]

Alcohol Intake and Risk of Stomach Cancer: A Pooled Analysis of 20 Cohorts

**Table S1.** Classification of Anatomical Subsites and Histological Subtypes of Stomach Cancer

**Figure S1.** Cohort- and Sex-specific Associations Between an Alcohol Intake of ≥30 g/day, Compared with 0.1–<5 g/day, and the Risk of Stomach Cancer.

**Figure S2.** Pooled Multivariable Hazard Ratios (HR) and 95% Confidence Intervals (95% CI) for Alcohol Intake and Risk of Stomach Cancer and Its Subtypes in Never Smokers

**Table S2.** Pooled Multivariable Hazard Ratios (HR)^a^ and 95% Confidence Intervals (95% CI) for Alcohol Intake and Risk of Subtypes of Stomach Cancer for Women

**Table S3.** Pooled Multivariable Hazard Ratios (HR)^a^ and 95% Confidence Intervals (95% CI) for Alcohol Intake and Risk of Subtypes of Stomach Cancer for Men

**Table S4.** The Role of *Helicobacter pylori* Infection Status in the Associations Between Alcohol Intake and Risk of Stomach Cancer

**Table S5.** Pooled Multivariable Hazard Ratios (HR)^a^ and 95% Confidence Intervals (95% CI) for Alcohol Intake and Risk of Stomach Cancer and Its Subtypes Excluding the First 2 Years of Follow-Up

**Table S6.** Pooled Multivariable Hazard Ratios (HR)^a^ and 95% Confidence Intervals (95% CI) for Alcohol Intake and Risk of Stomach Cancer in Cohorts Assessing Former Drinking

**Table S1.** Classification of Anatomical Subsites and Histological Subtypes of Stomach Cancer

| **Code** | **Description** |
| --- | --- |
| 151/C16 | Adenocarcinoma of the stomach |
| **Anatomical subsites (ICD-9/ICD-10)*** | |
| 151.0/C16.0 | Adenocarcinoma of the gastric cardia |
| 151.1-6/C16.1-6 | '' '' '' non-cardia |
| 151.3/C16.1 | '' '' '' fundus of stomach |
| 151.4/C16.2 | '' '' '' body of stomach |
| 151.5/C16.5 | '' '' '' lesser curvature of stomach |
| 151.6/C16.6 | '' '' '' greater curvature of stomach |
| 151.2/C16.3 | '' '' '' pyloric antrum |
| 151.1/C16.4 | '' '' '' pylorus |
| 151.8-9/C16.8-9 | '' '' '' overlapping or unspecified parts of the stomach |
| **Histological subtypes (ICD-O-3)†** | |
| Diffuse-type |  |
| 8141 | Scirrhous adenocarcinoma |
| 8142 | Linitis plastica |
| 8145 | Carcinoma, diffuse type |
| 8490 | Signet ring cell carcinoma |
| Intestinal-type |  |
| 8140 | Adenocarcinoma, NOS |
| 8143 | Superficial spreading adenocarcinoma |
| 8144 | Adenocarcinoma, intestinal type |
| 8210 | Adenocarcinoma in adenomatous polyp |
| 8211 | Tubular adenocarcinoma |
| 8221 | Adenocarcinoma in multiple adenomatous polyps |
| 8260 | Papillary adenocarcinoma |
| 8261 | Adenocarcinoma in villous adenoma |
| 8262 | Villous adenocarcinoma |
| 8263 | Adenocarcinoma in tubulovillous adenoma |
| Mixed, other or unknown type‡ |  |
| 8010 | Carcinoma, NOS |
| 8020 | Carcinoma, undifferentiated, NOS |
| 8021 | Carcinoma, anaplastic, NOS |
| 8190 | Trabecular adenocarcinoma |
| 8201 | Cribriform carcinoma, NOS |
| 8213 | Serrated adenocarcinoma |
| 8214 | Parietal cell adenocarcinoma |
| 8230 | Solid adenocarcinoma with mucin formation |
| 8255 | Adenocarcinoma with mixed subtypes |
| 8310 | Clear cell adenocarcinoma |
| 8320 | Granular cell adenocarcinoma |
| 8323 | Mixed cell adenocarcinoma |
| 8480 | Mucinous adenocarcinoma |
| 8481 | Mucin-producing adenocarcinoma |
| 8560 | Adenosquamous carcinoma |
| 8574 | Adenocarcinoma with neuroendocrine differentiation |
| 8576 | Hepatoid adenocarcinoma |

* Anatomical subsite classifications based on the 9^t^†† (ICD-9) or 10^t^†† version of the International Classification of Diseases (ICD-10).

† Histological subtype classification based on criteria from the International Classification of Diseases for Oncology (ICD-O-3).

‡ Not treated as study outcome for analysis.

**Figure S1.** Cohort- and Sex-specific Associations Between an Alcohol Intake of ≥30 g/day, Compared with 0.1–<5 g/day, and the Risk of Stomach Cancer.

Abbreviations: CNBSS: Canadian National Breast Screening Study; COSM: Cohort of Swedish Men; CPS-II: Cancer Prevention Study II Nutrition Cohort; EPIC: European Prospective Investigation into Cancer and Nutrition; HPFS: Health Professionals Follow-up Study; JPHC I: Japan Public Health Center-based Prospective Study I; JPHC II: Japan Public Health Center-based Prospective Study II; MCCS: Melbourne Collaborative Cohort Study; MEC: Multiethnic Cohort Study; NIH-AARP: NIH-AARP Diet and Health Study; NLCS: Netherlands Cohort Study; PLCO: Prostate, Lung, Colorectal, and Ovarian Cancer Screening Trial; SCHS: Singapore Chinese Health Study; SCS: Shanghai Cohort Study; SMHS: Shanghai Men’s Health Study; WHI, Women’s Health Initiative; VITAL: VITamins and Lifestyle Study: Cohort Study of Dietary Supplements and Cancer Risk; not included due to insufficient cases with alcohol intakes ≥30 g/day - IWHS: Iowa Women’s Health Study; NHS II: Nurses’ Health Study II; SMC: Swedish Mammography Cohort. f depicts female studies and m depicts male studies.

**Figure S2.** Pooled Multivariable Hazard Ratios (HR) and 95% Confidence Intervals (95% CI) for Alcohol Intake and Risk of Stomach Cancer and Its Subtypes in Never Smokers.

HRs and 95% CIs not reported when the number of cases is less than 10.

**Table S2.** Pooled Multivariable Hazard Ratios (HR)^a^ and 95% Confidence Intervals (95% CI) for Alcohol Intake and Risk of Subtypes of Stomach Cancer for Women

|  |  | **Per 10 g/day Increment** | ***P* value, Test for Common Effects by Subtype^b^** |
| --- | --- | --- | --- |
|  |  |  |  |
| By anatomical location† |  |  |  |
| Gastric cardia | No. of cases | 354 | .91^c^ |
|  | HR (95% CI) | 0.99 (0.90 to 1.08) |  |
| Non-cardia | No. of cases | 1,367 |  |
|  | HR (95% CI) | 0.99 (0.94 to 1.05) |  |
| Fundus | No. of cases | 83 |  |
|  | HR (95% CI) | 1.01 (0.90 to 1.14) |  |
| Body | No. of cases | 386 |  |
|  | HR (95% CI) | 1.07 (0.97 to 1.18) |  |
| Greater curvature | No. of cases | 60 |  |
|  | HR (95% CI) | 1.04 (0.97 to 1.11) |  |
| Lesser curvature | No. of cases | 163 |  |
|  | HR (95% CI) | 1.04 (0.91 to 1.19) |  |
| Pyloric antrum | No. of cases | 580 |  |
|  | HR (95% CI) | 0.92 (0.83 to 1.02) |  |
| Pylorus | No. of cases | 95 |  |
|  | HR (95% CI) | 0.72 (0.50 to 1.03) |  |
|  |  |  |  |
| By histological subtype± |  |  |  |
| Diffuse-type | No. of cases | 553 | .63^d^ |
|  | HR (95% CI) | 0.99 (0.91 to 1.08) |  |
| Intestinal-type | No. of cases | 1,690 |  |
|  | HR (95% CI) | 0.97 (0.91 to 1.02) |  |

^a^ Adjusted for race and ethnicity (Asian, Black, Hispanic, White, other), education (<high school, high school, >high school), cigarette smoking status (never, past, current), amount smoked (cigarettes/day, continuous), duration of cigarette smoking (years, continuous), body mass index (kg/m^2^, continuous), red meat intake (g/day, tertiles), fruit intake (g/day, tertiles) and total energy intake (kcal/day, continuous), stratified by age in years, year of questionnaire return and center (for European Prospective Investigation into Cancer and Nutrition). Analyses for continuous alcohol intake were further adjusted for an indicator variable identifying alcohol drinking status.

^b^ *P* value, test for common effects by subtype was calculated using a contrast test.

^c^ Test for common effects: gastric cardia *v* non-cardia cancers.

^d^ Test for common effects: diffuse-type *v* intestinal-type cancers.

† Gastric cardia: International Classification of Diseases (ICD) 9/ICD 10 codes 151.0/C16.0; non-cardia: 151.1-151.6/C16.1–C16.6; fundus of stomach: 151.3/C16.1; body of stomach: 151.4/C16.2; greater curvature of stomach: 151.6/C16.6; lesser curvature of stomach: 151.5/C16.5; pyloric antrum: 151.2/C16.3; pylorus: 151.1/C16.4; HRs not reported for overlapping/unspecified tumors (n=581): 151.8/C16.8 and 151.9/C16.9. These analyses do not include the Nurses’ Health Study as data on subsite were not available.

± Diffuse-type: International Classification of Diseases for Oncology codes 8141 scirrhous adenocarcinoma; 8142 linitis plastica; 8145 carcinoma, diffuse type; 8490 signet ring cell carcinoma; intestinal-type: 8140 adenocarcinoma, NOS; 8143 superficial spreading adenocarcinoma; 8144 adenocarcinoma, intestinal type; 8210 adenocarcinoma in adenomatous polyp; 8211 tubular adenocarcinoma; 8221 adenocarcinoma in multiple adenomatous polyps; 8260 papillary adenocarcinoma; 8261 adenocarcinoma in villous adenoma; 8262 villous adenocarcinoma; 8263 adenocarcinoma in tubulovillous adenoma; HRs not reported for mixed/other/unknown types (n=163): 8010 carcinoma, NOS; 8020 carcinoma, undifferentiated, NOS; 8021 carcinoma, anaplastic, NOS; 8190 trabecular adenocarcinoma; 8201 cribriform carcinoma, NOS; 8213 serrated adenocarcinoma; 8214 parietal cell adenocarcinoma; 8230 solid adenocarcinoma with mucin formation; 8255 adenocarcinoma with mixed subtypes; 8310 clear cell adenocarcinoma; 8320 granular cell adenocarcinoma; 8323 mixed cell adenocarcinoma; 8480 mucinous adenocarcinoma; 8481 mucin-producing adenocarcinoma; 8560 adenosquamous carcinoma; 8574 adenocarcinoma with neuroendocrine differentiation; 8576 hepatoid adenocarcinoma.

|  |  | **Per 10 g/day Increment** | ***P* value, Test for Common Effects by Subtype^b^** |
| --- | --- | --- | --- |
|  |  |  |  |
| By anatomical location† |  |  |  |
| Gastric cardia | No. of cases | 1,513 | .28^c^ |
|  | HR (95% CI) | 1.02 (1.00 to 1.05) |  |
| Non-cardia | No. of cases | 3,033 |  |
|  | HR (95% CI) | 1.01 (0.99 to 1.02) |  |
| Fundus | No. of cases | 209 |  |
|  | HR (95% CI) | 1.07 (1.02 to 1.12) |  |
| Body | No. of cases | 887 |  |
|  | HR (95% CI) | 1.02 (1.01 to 1.05) |  |
| Greater curvature | No. of cases | 93 |  |
|  | HR (95% CI) | 1.02 (0.94 to 1.10) |  |
| Lesser curvature | No. of cases | 431 |  |
|  | HR (95% CI) | 0.97 (0.92 to 1.02) |  |
| Pyloric antrum | No. of cases | 1,234 |  |
|  | HR (95% CI) | 1.00 (0.97 to 1.03) |  |
| Pylorus | No. of cases | 179 |  |
|  | HR (95% CI) | 0.98 (0.91 to 1.06) |  |
|  |  |  |  |
| By histological subtype± |  |  |  |
| Diffuse-type | No. of cases | 719 | .30^d^ |
|  | HR (95% CI) | 1.00 (0.96 to 1.04) |  |
| Intestinal-type | No. of cases | 4,814 |  |
|  | HR (95% CI) | 1.02 (1.00 to 1.03) |  |

**Table S3.** Pooled Multivariable Hazard Ratios (HR)^a^ and 95% Confidence Intervals (95% CI) for Alcohol Intake and Risk of Subtypes of Stomach Cancer for Men

^a^ Adjusted for race and ethnicity (Asian, Black, Hispanic, White, other), education (<high school, high school, >high school), cigarette smoking status (never, past, current), amount smoked (cigarettes/day, continuous), duration of cigarette smoking (years, continuous), body mass index (kg/m^2^, continuous), red meat intake (g/day, tertiles), fruit intake (g/day, tertiles) and total energy intake (kcal/day, continuous), stratified by age in years, year of questionnaire return and center (for European Prospective Investigation into Cancer and Nutrition). Analyses for continuous alcohol intake were further adjusted for an indicator variable identifying alcohol drinking status.

^b^ *P* value, test for common effects by subtype was calculated using a contrast test.

^c^ Test for common effects: gastric cardia *v* non-cardia cancers.

^d^ Test for common effects: diffuse-type *v* intestinal-type cancers.

† Gastric cardia: International Classification of Diseases (ICD) 9/ICD 10 codes 151.0/C16.0; non-cardia: 151.1-151.6/C16.1–C16.6; fundus of stomach: 151.3/C16.1; body of stomach: 151.4/C16.2; greater curvature of stomach: 151.6/C16.6; lesser curvature of stomach: 151.5/C16.5; pyloric antrum: 151.2/C16.3; pylorus: 151.1/C16.4; HRs not reported for overlapping/unspecified tumors (n=1,251): 151.8/C16.8 and 151.9/C16.9. These analyses do not include the Health Professionals Follow-up Study as data on subsite were not available.

± Diffuse-type: International Classification of Diseases for Oncology codes 8141 scirrhous adenocarcinoma; 8142 linitis plastica; 8145 carcinoma, diffuse type; 8490 signet ring cell carcinoma; intestinal-type: 8140 adenocarcinoma, NOS; 8143 superficial spreading adenocarcinoma; 8144 adenocarcinoma, intestinal type; 8210 adenocarcinoma in adenomatous polyp; 8211 tubular adenocarcinoma; 8221 adenocarcinoma in multiple adenomatous polyps; 8260 papillary adenocarcinoma; 8261 adenocarcinoma in villous adenoma; 8262 villous adenocarcinoma; 8263 adenocarcinoma in tubulovillous adenoma; HRs not reported for mixed/other/unknown types (n=418): 8010 carcinoma, NOS; 8020 carcinoma, undifferentiated, NOS; 8021 carcinoma, anaplastic, NOS; 8190 trabecular adenocarcinoma; 8201 cribriform carcinoma, NOS; 8213 serrated adenocarcinoma; 8214 parietal cell adenocarcinoma; 8230 solid adenocarcinoma with mucin formation; 8255 adenocarcinoma with mixed subtypes; 8310 clear cell adenocarcinoma; 8320 granular cell adenocarcinoma; 8323 mixed cell adenocarcinoma; 8480 mucinous adenocarcinoma; 8481 mucin-producing adenocarcinoma; 8560 adenosquamous carcinoma; 8574 adenocarcinoma with neuroendocrine differentiation; 8576 hepatoid adenocarcinoma. These analyses do not include the Health Professionals Follow-up Study as data on histology were not available.

**Table S4.** The Role of *Helicobacter pylori* Infection Serostatus in the Associations Between Alcohol Intake (per 10 g/day) and Risk of Stomach Cancer

|  | **Cases** | **Controls** | **Hazard Ratio/Odds Ratio (95% CI)** | ***P* value, Test for Interaction** |
| --- | --- | --- | --- | --- |
| Total stomach cancer |  |  |  |  |
| Pooled full cohort data |  |  |  |  |
| All participants | 3,253 | - | 1.02 (1.00 to 1.04)± |  |
| Nested case-control data# |  |  |  |  |
| All participants | 773 | 1,859 | 0.95 (0.91 to 1.00)±± |  |
| All participants (model adjusted for *Helicobacter pylori* status) | 773 | 1,859 | 0.96 (0.91 to 1.01)¶ |  |
| By *Helicobacter pylori* statu*s* |  |  |  | .43 |
| *Helicobacter pylori* positive | 670 | 1,265 | 0.95 (0.91 to 1.00)±± |  |
| *Helicobacter pylori* negative | 103 | 594 | 1.00 (0.90 to 1.11)±± |  |
|  |  |  |  |  |
| Gastric cardia† |  |  |  |  |
| Pooled full cohort data |  |  |  |  |
| All participants | 456 | - | 1.00 (0.95 to 1.04)± |  |
| Nested case-control data# |  |  |  |  |
| All participants | 151 | 388 | 0.94 (0.85 to 1.06)±± |  |
| All participants (model adjusted for *Helicobacter pylori* status) | 151 | 388 | 0.95 (0.85 to 1.06)¶ |  |
| By *Helicobacter pylori* statu*s* |  |  |  | .67 |
| *Helicobacter pylori* positive | 92 | 215 | 0.93 (0.81 to 1.07)±± |  |
| *Helicobacter pylori* negative | 59 | 173 | 0.97 (0.83 to 1.13)±± |  |
|  |  |  |  |  |
| Non-cardia† |  |  |  |  |
| Pooled full cohort data |  |  |  |  |
| All participants | 2,042 | - | 1.02 (1.00 to 1.04)± |  |
| Nested case-control data# |  |  |  |  |
| All participants | 407 | 873 | 0.97 (0.91 to 1.03)±± |  |
| All participants (model adjusted for *Helicobacter pylori* status) | 407 | 873 | 0.98 (0.91 to 1.04)¶ |  |
| By *Helicobacter pylori* statu*s* |  |  |  | .22 |
| *Helicobacter pylori* positive | 388 | 662 | 0.97 (0.91 to 1.04)±± |  |
| *Helicobacter pylori* negative | 19 | 211 | 1.12 (0.90 to 1.38)±± |  |
|  |  |  |  |  |

All analyses included data from the European Prospective Investigation into Cancer and Nutrition, Japan Public Health Center-based Prospective Study I, Japan Public Health Center-based Prospective Study II, Melbourne Collaborative Cohort Study, Prostate, Lung, Colorectal, and Ovarian Cancer Screening Trial (men only) and Shanghai Men’s Health Study.

± Sex-specific cohorts were analyzed. Adjusted for race and ethnicity (Asian, Black, Hispanic, White, other), education (<high school, high school, >high school), cigarette smoking status (never, past, current), amount smoked (cigarettes/day, continuous), duration of cigarette smoking (years, continuous), body mass index (kg/m^2^, continuous), total red meat intake (g/day, quintiles), fruit intake (g/day, quintiles), total energy (kcal/day, continuous) and an indicator variable denoting alcohol intake as any or none to account for former drinkers, stratified by age in years, year of questionnaire return and center (for European Prospective Investigation into Cancer and Nutrition).

\# Cases and controls were matched using a combination of these factors in individual studies: age, sex, race and ethnicity, country of birth/center (EPIC), date of blood collection, fasting time at blood collection and antibiotic use in the last week.

±± Adjusted for race and ethnicity (Asian, Black, Hispanic, White, other), education (<high school, high school, >high school), cigarette smoking status (never, past, current), amount smoked (cigarettes/day, continuous), duration of cigarette smoking (years, continuous), body mass index (kg/m^2^, continuous), red meat intake (g/day, quintiles), fruit intake (g/day, quintiles), total energy intake (kcal/day, continuous) and an indicator variable denoting alcohol intake as any or none to account for former drinkers.

¶ Additionally adjusted for *Helicobacter pylori* infection status.

† Gastric cardia: International Classification of Diseases (ICD) 9/ICD 10 codes 151.0/C16.0; non-cardia: 151.1-151.6/C16.1–C16.6.

**Table S5.** Pooled Multivariable Hazard Ratios (HR)^a^ and 95% Confidence Intervals (95% CI) for Alcohol Intake and Risk of Stomach Cancer and Its Subtypes Excluding the First 2 Years of Follow-Up

|  |  | **Per 10 g/day Increment** | ***P* value, Test for Common Effects by Subtype^b^** | **Alcohol Intake Categories (g/day)** | | | | | ***P* trend^d^** | ***P* value, Test for Common Effects by Subtype, Highest Category^b^** |
| --- | --- | --- | --- | --- | --- | --- | --- | --- | --- | --- |
|  |  |  |  | **Abstention^c^** | **0.1–<5** | **5–<15** | **15–<30** | **≥30** |  |  |
| Stomach cancer | No. of cases (%) | 7,645 (100) |  | 2,985 (39.1) | 1,509 (19.7) | 1,086 (14.2) | 887 (11.6) | 1,178 (15.4) |  |  |
|  | HR (95% CI) | 1.01 (0.99 to 1.02) |  | 1.06 (0.98 to 1.15) | 1.00 (ref) | 0.95 (0.87 to 1.03) | 1.01 (0.89 to 1.14) | 1.05 (0.95 to 1.16) | .28 |  |
|  |  |  |  |  |  |  |  |  |  |  |
| By anatomical location† |  |  |  |  |  |  |  |  |  |  |
| Gastric cardia | No. of cases (%) | 1,671 (100) | .68^e^ | 498 (29.8) | 396 (23.7) | 302 (18.1) | 231 (13.8) | 244 (14.6) |  | .70^e^ |
|  | HR (95% CI) | 1.02 (0.99 to 1.04) |  | 1.13 (0.98 to 1.30) | 1.00 (ref) | 1.01 (0.87 to 1.18) | 1.05 (0.89 to 1.29) | 1.06 (0.89 to 1.25) | .49 |  |
| Non-cardia | No. of cases (%) | 4,089 (100) |  | 1,779 (43.5) | 705 (17.3) | 488 (11.9) | 442 (10.8) | 675 (16.5) |  |  |
|  | HR (95% CI) | 1.01 (1.00 to 1.03) |  | 1.02 (0.93 to 1.12) | 1.00 (ref) | 0.95 (0.85 to 1.07) | 1.02 (0.90 to 1.15) | 1.10 (0.98 to 1.24) | .0004 |  |
| Fundus | No. of cases (%) | 264 (100) |  | 102 (38.6) | 39 (14.8) | 35 (13.3) | 34 (12.9) | 54 (20.4) |  |  |
|  | HR (95% CI) | 1.07 (1.02 to 1.12) |  | 1.51 (1.02 to 2.24) | 1.00 (ref) | 1.26 (0.79 to 2.00) | 1.51 (0.93 to 2.45) | 1.62 (1.04 to 2.55) | .04 |  |
| Body | No. of cases (%) | 1,212 (100) |  | 525 (43.3) | 169 (13.9) | 139 (11.5) | 122 (10.1) | 257 (21.2) |  |  |
|  | HR (95% CI) | 1.04 (1.01 to 1.06) |  | 1.20 (0.99 to 1.45) | 1.00 (ref) | 1.06 (0.84 to 1.34) | 1.07(0.84 to 1.34) | 1.42 (1.14 to 1.77) | .0004 |  |
| Greater curvature | No. of cases (%) | 138 (100) |  | 43 (31.1) | 35 (25.4) | 20 (14.5) | 15 (10.9) | 25 (18.1) |  |  |
|  | HR (95% CI) | 1.05 (0.98 to 1.12) |  | 0.59 (0.36 to 0.94) | 1.00 (ref) | 0.97 (0.55 to 1.70) | 1.12 (0.59 to 2.13) | 1.67 (0.92 to 3.03) | .07 |  |
| Lesser curvature | No. of cases (%) | 522 (100) |  | 239 (45.8) | 98 (18.8) | 60 (11.5) | 63 (12.0) | 62 (11.9) |  |  |
|  | HR (95% CI) | 0.99 (0.94 to 1.04) |  | 1.00 (0.77 to 1.28) | 1.00 (ref) | 0.87 (0.63 to 1.21) | 1.13 (0.81 to 1.59) | 1.00 (0.71 to 1.42) | .75 |  |
| Pyloric antrum | No. of cases (%) | 1,697 (100) |  | 786 (46.3) | 285 (16.8) | 198 (11.7) | 183 (10.8) | 245 (14.4) |  |  |
|  | HR (95% CI) | 0.99 (0.96 to 1.02) |  | 0.99 (0.86 to 1.15) | 1.00 (ref) | 0.94 (0.78 to 1.13) | 0.96 (0.79 to 1.16) | 0.92 (0.76 to 1.11) | .46 |  |
| Pylorus | No. of cases (%) | 256 (100) |  | 84 (32.8) | 79 (30.8) | 36 (14.1) | 25 (9.8) | 32 (12.5) |  |  |
|  | HR (95% CI) | 0.97 (0.90 to 1.06) |  | 0.68 (0.48 to 0.96) | 1.00 (ref) | 0.70 (0.46 to 1.04) | 0.73 (0.46 to 1.18) | 0.71 (0.45 to 1.12) | .21 |  |
|  |  |  |  |  |  |  |  |  |  |  |
| By histological subtype± |  |  |  |  |  |  |  |  |  |  |
| Diffuse-type | No. of cases (%) | 1,150 (100) | .56^f^ | 460 (40.0) | 267 (23.2) | 166 (14.4) | 126 (11.0) | 131 (11.4) |  | .52^f^ |
|  | HR (95% CI) | 1.00 (0.96 to 1.03) |  | 1.02 (0.86 to 1.19) | 1.00 (ref) | 0.97 (0.79 to 1.18) | 1.00 (0.80 to 1.251) | 1.00 (0.80 to 1.26) | .37 |  |
| Intestinal-type | No. of cases (%) | 5,974 (100) |  | 2,324 (38.9) | 1,128 (18.9) | 839 (14.0) | 706 (11.8) | 977 (16.4) |  |  |
|  | HR (95% CI) | 1.01 (1.00 to 1.03) |  | 1.07 (0.99 to 1.15) | 1.00 (ref) | 0.96 (0.88 to 1.05) | 1.03 (0.94 to 1.14) | 1.09 (0.99 to 1.19) | .03 |  |

^a^ Sex-specific cohorts were analyzed. Adjusted for race and ethnicity (Asian, Black, Hispanic, White, other), education (<high school, high school, >high school), cigarette smoking status (never, past, current), amount smoked (cigarettes/day, continuous), duration of cigarette smoking (years, continuous), body mass index (kg/m^2^, continuous), red meat intake (g/day, tertiles), fruit intake (g/day, tertiles) and total energy intake (kcal/day, continuous), stratified by age in years, year of questionnaire return and center (for European Prospective Investigation into Cancer and Nutrition). Analyses for continuous alcohol intake were further adjusted for an indicator variable identifying alcohol drinking status.

^b^ *P* value, test for common effects by subtype was calculated using a contrast test.

^c^ Abstention was defined as <0.1 g/day.

^d^ *P* value, test for trend was calculated in drinkers (intake ≥0.1 g/day) using the Wald test statistic with the median of each intake category fit as a continuous variable.

^e^ Test for common effects: gastric cardia *v* non-cardia cancers.

^f^ Test for common effects: diffuse-type *v* intestinal-type cancers.

† Gastric cardia: International Classification of Diseases (ICD) 9/ICD 10 codes 151.0/C16.0; non-cardia: 151.1-151.6/C16.1–C16.6; fundus of stomach: 151.3/C16.1; body of stomach: 151.4/C16.2; greater curvature of stomach: 151.6/C16.6; lesser curvature of stomach: 151.5/C16.5; pyloric antrum: 151.2/C16.3; pylorus: 151.1/C16.4; HRs not reported for overlapping/unspecified tumors (n=1,627): 151.8/C16.8 and 151.9/C16.9. These analyses do not include the Health Professionals Follow-up Study or Nurses’ Health Study as data on subsite were not available.

± Diffuse-type: International Classification of Diseases for Oncology codes 8141 scirrhous adenocarcinoma; 8142 linitis plastica; 8145 carcinoma, diffuse type; 8490 signet ring cell carcinoma; intestinal-type: 8140 adenocarcinoma, NOS; 8143 superficial spreading adenocarcinoma; 8144 adenocarcinoma, intestinal type; 8210 adenocarcinoma in adenomatous polyp; 8211 tubular adenocarcinoma; 8221 adenocarcinoma in multiple adenomatous polyps; 8260 papillary adenocarcinoma; 8261 adenocarcinoma in villous adenoma; 8262 villous adenocarcinoma; 8263 adenocarcinoma in tubulovillous adenoma; HRs not reported for mixed/other/unknown types (n=376): 8010 carcinoma, NOS; 8020 carcinoma, undifferentiated, NOS; 8021 carcinoma, anaplastic, NOS; 8190 trabecular adenocarcinoma; 8201 cribriform carcinoma, NOS; 8213 serrated adenocarcinoma; 8214 parietal cell adenocarcinoma; 8230 solid adenocarcinoma with mucin formation; 8255 adenocarcinoma with mixed subtypes; 8310 clear cell adenocarcinoma; 8320 granular cell adenocarcinoma; 8323 mixed cell adenocarcinoma; 8480 mucinous adenocarcinoma; 8481 mucin-producing adenocarcinoma; 8560 adenosquamous carcinoma; 8574 adenocarcinoma with neuroendocrine differentiation; 8576 hepatoid adenocarcinoma. These analyses do not include the Health Professionals Follow-up Study as data on histology were not available.

**Table S6.** Pooled Multivariable Hazard Ratios (HR)^a^ and 95% Confidence Intervals (95% CI) for Alcohol Intake and Risk of Stomach Cancer in Cohorts Assessing Former Drinking

|  | **No. of cases (%)** | **HR (95% CI) without separating out former drinking** | **HR (95% CI) with former drinking separated out** |
| --- | --- | --- | --- |
| Total stomach |  |  |  |
| Former drinking | 345 (21.0) | --- | 0.99 (0.74 to 1.33) |
| Abstention^b^ | 376 (22.8) | 1.00 (0.86 to 1.16) | 0.96 (0.81 to 1.15) |
| 0.1 - <5 g/day | 482 (29.3) | 1.00 (ref) | 1.00 (ref) |
| 5 - <15 g/day | 230 (14.0) | 0.90 (0.78 to 1.04) | 0.90 (0.78 to 1.04) |
| ≥15 g/day | 212 (12.9) | 0.95 (0.82 to 1.11) | 0.95 (0.81 to 1.10) |

This analysis included data from the Cohort of Swedish Men, European Prospective Investigation into Cancer and Nutrition, Health Professionals Follow-up Study, Melbourne Collaborative Cohort Study, Nurses’ Health Study, Prostate, Lung, Colorectal, and Ovarian Cancer Screening Trial and Swedish Mammography Cohort.

^a^ Sex-specific cohorts were analyzed. Adjusted for race and ethnicity (Asian, Black, Hispanic, other, White), education (<high school, high school, >high school), cigarette smoking status (never, past, current), amount smoked (cigarettes/day, continuous), duration of cigarette smoking (years, continuous), body mass index (kg/m^2^, continuous), red meat intake (g/day, quintiles), fruit intake (g/day, quintiles) and total energy intake (kcal/day, continuous), stratified by age in years, year of questionnaire return and center (for European Prospective Investigation into Cancer and Nutrition).

^c^ Abstention was defined as <0.1 g/day.
